# Biomimetic Deposition of Zn-Doped Calcium Phosphate Coatings on Surface-Activated Ti6Al4V for Multifunctional Implant Interfaces

**DOI:** 10.3390/jfb17050225

**Published:** 2026-05-02

**Authors:** Yaimi Martín-Santana, Yadira González-Carranza, Leonel Díaz-Tato, Arturo Juárez-Hernández, Edgar Omar García-Sánchez, Myriam Angélica De La Garza-Ramos, Edén Amaral Rodríguez-Castellanos, Marco Antonio Loudovic Hernández-Rodríguez

**Affiliations:** 1 Facultad de Ingeniería Mecánica y Eléctrica (FIME), Universidad Autónoma de Nuevo León (UANL), San Nicolás de los Garza 66450, Nuevo León, Mexico; yaimi.martinstn@uanl.edu.mx (Y.M.-S.); yadira.gonzalezcn@uanl.edu.mx (Y.G.-C.); leonel.diazat@uanl.edu.mx (L.D.-T.); arturo.juarezhn@uanl.edu.mx (A.J.-H.); edgar.garciasc@uanl.edu.mx (E.O.G.-S.); 2 MAGEOTEC, Frutilla 214, Mirasol 1o., Monterrey 64102, Nuevo León, Mexico; 3 Unidad de Odontología Integral y Especialidades, Centro de Investigación y Desarrollo en Ciencias de la Salud (CIDICS), Universidad Autónoma de Nuevo León (UANL), Ave. Carlos Canseco s/n con Ave. Gonzalitos, Mitras Centro, Monterrey 64460, Nuevo León, Mexico; myriam.garzarm@uanl.edu.mx; 4 Departamento de Microbiología, Facultad de Odontología, Universidad Autónoma de Nuevo León (UANL), Monterrey 64460, Nuevo León, Mexico

**Keywords:** biomimetic calcium phosphate coatings, Zinc-doped apatite, Ti6Al4V surface modification, antibacterial implant surfaces, controlled Zn^2+^ ion release, osteointegrative multifunctional coatings

## Abstract

The design of implant surfaces that support bone integration while limiting bacterial colonization remains a central challenge in biomaterials science and engineering. In this work, zinc-doped biomimetic calcium phosphate (CaP-Zn) coatings were fabricated on Ti6Al4V through surface activation followed by deposition in supersaturated simulated body fluid (SBF). Acid and alkali–calcium treatments produced a porous, calcium-rich interface that enabled the uniform formation of apatite-like CaP layers. Zinc incorporation was achieved without suppressing the formation of CaP phases and led to systematic changes in coating microstructure and surface chemistry. Spectroscopic and structural analyses indicated Zn incorporation within the CaP matrix, consistent with partial Ca^2+^ substitution and its association with poorly crystalline domains. These features promoted controlled ionic release and localized dissolution–reprecipitation behavior. Antibacterial testing against *Streptococcus mutans* revealed a clear Zn-dependent reduction in bacterial viability, while cytocompatibility remained within acceptable limits at moderate Zn levels. Finally, the coatings combine intrinsic bioactivity with ion-mediated antibacterial functionality, offering a multifunctional surface strategy for advanced titanium-based implants.

## 1. Introduction

The demand for load-bearing biomedical implants has increased steadily over recent decades due to population aging and the growing prevalence of degenerative musculoskeletal diseases [1]. Metallic biomaterials, particularly titanium and its alloys, are widely used in orthopedic and dental applications because of their favorable mechanical strength, fatigue resistance, corrosion stability in physiological environments, and excellent biocompatibility [2]. Among these, Ti6Al4V remains the most extensively employed alloy for load-bearing implants due to its balance between mechanical performance and biological acceptance [3].

Despite these advantages, titanium and its alloys are considered bioinert materials and do not form a direct chemical bond with bone tissue. As a result, their intrinsic osteoconductive capacity is limited, and bone–implant integration relies primarily on mechanical interlocking rather than true biological bonding [4]. Insufficient or delayed osseointegration may compromise primary stability and long-term implant performance, particularly in patients with low bone quality or in applications requiring rapid functional loading [5].

To overcome these limitations, extensive research efforts have focused on modifying implant surfaces to enhance their bioactivity and promote faster and stronger bone–implant bonding [6]. Surface modification strategies include physical, chemical, and biochemical approaches, among which the application of bioactive ceramic coatings has proven particularly effective [7]. Calcium phosphate (CaP) ceramics, especially hydroxyapatite (HA, Ca_10_(PO_4_)_6_(OH)_2_), are widely used due to their close chemical and crystallographic similarity to the mineral phase of natural bone [8]. HA coatings have been shown to promote osteoconduction, accelerate bone apposition, and improve the interfacial bonding strength between bone and metallic substrates [9].

Several coating techniques have been developed for depositing CaP and HA layers on titanium substrates, including plasma spraying, sol–gel processing, electrophoretic deposition, electrochemical methods, and physical vapor deposition [10]. However, many of these approaches involve high processing temperatures, resulting in non-uniform microstructures, poor interfacial adhesion, or limited control over coating composition and crystallinity [11,12]. These drawbacks may negatively affect coating stability, long-term performance, and biological response.

In this context, the biomimetic coating method, originally proposed by Kokubo and co-workers, has emerged as a versatile and low-temperature alternative for producing bioactive CaP coatings under physiologically relevant conditions [13]. This method relies on the immersion of substrates in simulated body fluid (SBF), which mimics the ionic composition of human blood plasma, enabling the nucleation and growth of bone-like apatite layers at 37 °C and near-neutral pH [13]. Systematic studies have demonstrated that the formation of apatite in SBF correlates with in vivo bone-bonding ability, making this approach a valuable tool for evaluating and inducing bioactivity [13,14].

Subsequent refinements of the biomimetic process, including the use of concentrated SBF solutions (e.g., 5× and 10× SBF), have significantly reduced coating times and enabled better control over coating thickness, phase composition, and crystallinity [15]. It has been demonstrated in the literature that ionic strength, carbonate concentration, and solution supersaturation strongly influence CaP nucleation kinetics, coating morphology, and phase evolution [16]. These parameters are critical for tailoring coating properties relevant to biological performance. However, while bioactive CaP coatings significantly enhance osseointegration, they may also unintentionally promote bacterial adhesion and biofilm formation on implant surfaces [17]. Postoperative infections associated with implantable medical devices remain one of the most serious complications in orthopedic and dental surgery and are a leading cause of implant failure [18]. Bacterial colonization can occur within minutes after implantation, initiating a competitive process between host cell attachment and microbial adhesion, often described as the “race for the surface” [19].

Biofilms are structured microbial communities embedded in a self-produced extracellular polymeric matrix that confers strong resistance to antibiotics and host immune responses [20]. Once established, biofilms are extremely difficult to eradicate and frequently require surgical implant removal, leading to increased patient morbidity and healthcare costs [21]. Conventional systemic antibiotic therapies often fail to achieve sufficient local concentrations at the bone–implant interface and may contribute to the emergence of antibiotic-resistant strains [22]. To address this challenge, recent strategies have focused on developing multifunctional implant surfaces that simultaneously promote osseointegration while inhibiting bacterial colonization [23]. One promising approach involves the incorporation of antibacterial inorganic ions into bioactive CaP and HA coatings [24]. Among these, zinc (Zn^2+^) has attracted considerable attention due to its dual role in bone metabolism and antimicrobial activity [25].

Zinc is an essential trace element in bone tissue and plays a crucial role in osteoblast proliferation, osteogenic differentiation, and suppression of osteoclastic bone resorption [26]. In addition, Zn^2+^ exhibits broad-spectrum antibacterial activity against both Gram-positive and Gram-negative bacteria and has been shown to reduce inflammatory responses [27]. Several studies have extensively reviewed the structural and biological effects of ionic substitutions in calcium phosphates, highlighting the influence of Zn^2+^ on crystallinity, solubility, and biological behavior [28]. Other studies have emphasized the potential of ion-doped calcium phosphates for imparting multifunctional properties, including antimicrobial activity and controlled ion release [29].

Despite these advantages, the biological and structural effects of zinc incorporation into CaP and HA systems remain highly dependent on concentration, substitution mechanism, and processing route [30]. Several studies have reported dose-dependent antibacterial efficacy of Zn-substituted HA powders [31]; however, reduced antibacterial performance has often been observed when Zn-HA is applied in coating form, likely due to altered ion release kinetics and coating crystallinity [32]. Some studies have further emphasized that excessive Zn incorporation may negatively affect coating crystallinity and potentially induce cytotoxic responses, highlighting the need for careful optimization [33].

Moreover, the majority of Zn-containing CaP coatings reported in the literature have been produced using high-temperature or electrochemical methods, which may suffer from adhesion issues and compositional inhomogeneity [34]. Recent advances in surface engineering have increasingly focused on the development of multifunctional coatings capable of simultaneously addressing mechanical stability, corrosion behavior, and biological performance. In this context, studies on plasma electrolytic oxidation (PEO) coatings, particularly in magnesium-based systems, have demonstrated the potential of designing gradient and composite architectures that couple controlled degradation with the release of therapeutic species. These approaches highlight the importance of tailoring coating microstructure and composition to achieve a synergistic relationship between degradation kinetics and biological functionality, including ion-mediated antibacterial effects and tissue regeneration. Although such strategies have been predominantly explored in biodegradable metals, the underlying principles (namely, the integration of controlled ion release, hierarchical coating structures, and functional responsiveness) are directly applicable to titanium-based implant systems. Therefore, these developments provide a valuable conceptual framework for the present study, where biomimetic Zn–CaP coatings are designed to achieve a similar balance between bioactivity and antibacterial performance through controlled Zn^2+^ incorporation and release under physiologically relevant conditions [35,36]. In contrast, the biomimetic method offers a unique opportunity to incorporate Zn^2+^ directly during apatite nucleation and growth, enabling homogeneous ion distribution and controlled release under mild processing conditions [37].

Despite the extensive research on Zn-doped CaP and HA coatings, several limitations remain in current approaches, particularly those based on high-temperature or electrochemical techniques. These methods often result in heterogeneous Zn distribution, limited control over the physicochemical state of the dopant, residual stresses, and restricted tunability of ion release under physiologically relevant conditions. In this context, the present work addresses a critical gap by employing a biomimetic deposition strategy that enables the in situ incorporation of Zn^2+^ during apatite nucleation and growth, rather than post-synthesis doping. This approach provides enhanced control over the distribution and chemical state of Zn, promoting the coexistence of structurally incorporated and surface-accessible species, which in turn leads to a dual-stage release behavior characterized by an initial antibacterial effect followed by sustained ion delivery. Furthermore, the low-temperature processing conditions (~37 °C) favor the formation of carbonate-containing, poorly crystalline apatite phases that closely resemble biological mineral, thereby improving reactivity and bioactivity. Unlike previous studies that often evaluate either antibacterial or biological performance independently, this work establishes a direct correlation between coating structure, Zn incorporation, ion release kinetics, antibacterial activity, and cytocompatibility. Importantly, the results demonstrate that Zn release is governed not only by its concentration but by its physicochemical state and spatial distribution within the coating, providing new insight into the design of multifunctional implant surfaces. Thus, the biomimetic method is not only a processing alternative but a key enabler for achieving controlled ion delivery, improved interfacial integration, and biologically relevant coating functionality.

Based on these considerations, the development of biomimetic Zn-doped calcium phosphate (Zn–CaP) coatings on surface-activated Ti6Al4V substrates represents a promising strategy to simultaneously address the challenges of rapid osseointegration and infection prevention.

Accordingly, the aim of this study is to develop and systematically evaluate Zn–CaP coatings produced via biomimetic deposition, with particular emphasis on (i) the role of surface activation in promoting coating nucleation, (ii) the influence of Zn incorporation on coating morphology, phase composition, and crystallinity, and (iii) the relationship between Zn physicochemical state, ion release kinetics, antibacterial performance, and cytocompatibility. The central hypothesis is that controlled in situ incorporation of Zn^2+^ during biomimetic apatite formation enables the design of multifunctional coatings capable of delivering a balanced biological response, combining bioactivity, antibacterial efficacy, and cytocompatibility at the bone–implant interface.

## 2. Materials and Methods

### 2.1. Substrate Material and Sample Preparation

Commercial Ti6Al4V ELI alloy bars were used as the substrate material in this study. The material was supplied by United Performance Metals Inc., Hamilton, OH, USA. Cylindrical specimens were machined using a CNC micromachining lathe (Nanoform X, Moore Nanotechnology Systems, Keene, NH, USA) to obtain discs with a diameter of 6.35 mm and a thickness of 3.50 mm. To remove residual cutting fluids, oils, and surface contaminants introduced during machining, all specimens were subjected to a standardized cleaning protocol. Samples were first washed in a 2 wt.% Alconox detergent solution (Alconox Inc., White Plains, NY, USA) for 15 min using a rotary washing system. Subsequently, specimens were thoroughly rinsed with running distilled water to eliminate detergent residues. Final cleaning was performed by ultrasonic treatment in reagent-grade distilled water for 5 min. All experiments were conducted using reagent-grade distilled water (CTR Scientific, Monterrey, NL, Mexico). After cleaning, samples were dried using a stream of warm air and stored in a clean environment prior to surface treatments.

### 2.2. Acid Etching Treatment

Surface modification by acid etching was performed following a procedure adapted from Wen et al. [38]. The Ti6Al4V specimens were immersed in a mixed acid solution composed of hydrochloric acid (HCl) and sulfuric acid (H_2_SO_4_) at a volumetric ratio of 1:1, corresponding to an overall acid concentration of 67%. The etching treatment was carried out at 80 °C for 20 min under static conditions. The acids were supplied by CTR Scientific, Monterrey, NL, Mexico. This process was designed to remove the native oxide layer, generate microscale surface roughness, and introduce surface porosity. Upon completion of the etching period, specimens were removed from the acid solution using plastic tweezers and immediately rinsed with copious amounts of distilled water to stop the reaction and remove residual acids. Samples were subsequently dried at room temperature under ambient conditions.

Alternative surface modification methods were carefully considered, including single-acid treatments (e.g., HCl or H_2_SO_4_ alone), alkaline treatments (e.g., NaOH), and physical methods such as sandblasting or grit blasting. However, these approaches present certain limitations depending on the intended application. For instance, single-acid treatments often result in less homogeneous and less reactive surfaces, while alkaline treatments tend to form sodium titanate layers that require additional heat treatment to become bioactive and are typically used in bioactive glass-like surface formation, not immediate CaP nucleation. Mechanical methods (e.g., grit blasting), although effective in increasing roughness, may introduce surface contamination (e.g., alumina residues) or residual stresses and do not provide the same level of chemical activation, which can negatively affect coating uniformity and reproducibility.

In contrast, the combined HCl/H_2_SO_4_ etching at elevated temperature promotes the formation of micro- and submicron-scale roughness, while simultaneously increasing surface energy and hydroxyl group density. Crucially, in the present work, the acid etching step is not an isolated treatment, but part of a multi-step surface activation strategy (acid → alkali → Ca^2+^ exchange → thermal → water treatment). The initial HCl/H_2_SO_4_ etching plays a determinant role in enabling the subsequent formation of a reactive titanate layer, which ultimately governs the nucleation kinetics, adhesion, and uniformity of the biomimetically deposited CaP coatings. This sequential synergy is widely recognized as essential for achieving highly bioactive titanium surfaces [39,40,41,42,43].

The selection of the HCl/H_2_SO_4_ etching treatment at 80 °C for 20 min is firmly grounded in established surface engineering protocols for Ti6Al4V alloys, particularly in the context of biomimetic calcium phosphate (CaP) deposition and bioactive implant interfaces.

The use of mixed strong acids (HCl/H_2_SO_4_) has been consistently demonstrated to be significantly more effective than single-acid systems in generating a hierarchically roughened and chemically active titanium surface. This is attributed to the synergistic effect between chloride ions (promoting localized dissolution) and sulfate ions (favoring controlled oxidation–reprecipitation phenomena), which together produce a uniform micro/submicron topography and high density of Ti–OH functional groups (both critical for nucleation of CaP phases). It has been demonstrated that dual-acid treatments produce more homogeneous and bioactive surfaces compared to single-acid etching [44,45]. Similarly, in the literature, it was reported that mixed-acid treatments significantly enhance apatite-forming ability due to increased surface hydroxylation and surface energy [46,47,48].

The temperature of 80 °C is a crucial parameter, not merely a processing convenience. Elevated temperature accelerates the dissolution–reprecipitation kinetics, enabling the formation of well-defined microcavities and increased surface area within controlled timeframes, while avoiding excessive or uncontrolled corrosion. This has been validated in multiple studies where temperatures in the range of 60–90 °C were identified as optimal for achieving reproducible surface activation without compromising the mechanical integrity of Ti6Al4V [49,50]. Lower temperatures typically result in insufficient activation, whereas excessively high temperatures or prolonged exposure can induce detrimental surface weakening.

Regarding etching duration (20 min), this parameter was carefully selected and experimentally validated to strike a balance between: (i) effective removal of the native TiO_2_ passive layer, (ii) generation of controlled micro-roughness, and (iii) preservation of substrate integrity. Over-etching is well known to produce irregular morphologies, excessive material loss, and even microcrack initiation, which can adversely affect coating adhesion and long-term implant performance [51,52]. Therefore, the selected duration aligns with optimized conditions reported in the literature and confirmed through our preliminary trials.

### 2.3. Surface Activation by Alkali and Calcium Treatments

Following acid etching, the specimens were subjected to a multi-step surface activation procedure involving alkaline treatment, calcium ion exchange, thermal treatment, and water immersion. All reagents in this stage were supplied by Sigma-Aldrich (now Merck), St. Louis, MO, USA.

#### 2.3.1. Alkali Treatment

A 10 M NaOH solution was prepared by dissolving 40 g of NaOH pellets in 100 mL of distilled water. The solution was heated to 60 °C using a hot plate equipped with temperature control. Each specimen was immersed individually in glass tubes containing 5 mL of the NaOH solution and maintained under these conditions for 24 h. After completion of the alkali treatment, the samples were removed and thoroughly rinsed with distilled water to eliminate residual NaOH and reaction by-products.

#### 2.3.2. Calcium Treatment

A 1 M CaCl_2_ solution was prepared by dissolving 11.1 g of CaCl_2_ in 100 mL of distilled water. The alkali-treated specimens were immersed individually in the CaCl_2_ solution at 40 °C for 24 h to promote Na^+^/Ca^2+^ ion exchange and formation of calcium-containing titanate species. After treatment, specimens were rinsed with distilled water and dried at room temperature.

#### 2.3.3. Thermal Treatment and Water Immersion

The samples were subsequently subjected to thermal treatment in a muffle furnace (Thermo Fisher Scientific, Waltham, MA, USA) at 600 °C for 1 h to stabilize the surface layer and promote partial crystallization of titanate phases. After thermal treatment, specimens were allowed to cool slowly inside the furnace to room temperature. Finally, the samples were immersed in deionized water at 80 °C for 24 h (denoted as H_2_O treatment). This step was applied to further hydroxylate the surface and enhance the formation of Ti–OH functional groups. After water treatment, the samples were rinsed with distilled water and dried at room temperature.

### 2.4. Biomimetic Deposition of Calcium Phosphate (CaP) Coatings

#### 2.4.1. Preparation of Supersaturated CaP Solutions

Biomimetic CaP coatings were deposited using modified supersaturated solutions [53]. Two solution variants were prepared by increasing the calcium ion concentration to 7 and 10 times that of conventional simulated body fluid (SBF), referred to as SBFX7 and SBFX10, respectively. Calcium chloride (CaCl_2_), monosodium phosphate (NaH_2_PO_4_), and sodium bicarbonate (NaHCO_3_) were used as sources of calcium, phosphate, and carbonate ions, respectively [54]. All reagents used in this stage were supplied by Sigma-Aldrich (Merck, St. Louis, MO, USA). The mass and molar concentrations of the salts used to prepare 500 mL of each solution are summarized in Table 1.

#### 2.4.2. Coating Deposition Procedure

For coating deposition, 500 mL of distilled water (CTR Scientific, Monterrey, NL, Mexico) was placed in a glass beaker and heated to 36.5 °C on a hot plate with continuous magnetic stirring at 100 rpm. CaCl_2_ and NaH_2_PO_4_ were added sequentially, with a 5 min interval between additions to allow partial equilibration. Five minutes after the addition of the last salt, the activated Ti6Al4V specimens were immersed in the solution. After 30 min from the final salt addition, NaHCO_3_ was added to introduce carbonate species and adjust the solution chemistry. Temperature and stirring conditions were maintained constant throughout the deposition period. At the end of the deposition time, specimens were removed from the solution, rinsed with deionized water (CTR Scientific, Monterrey, NL, Mexico), and dried at room temperature.

#### 2.4.3. Deposition Variants

To evaluate the influence of solution concentration and immersion time, four deposition conditions were investigated, as summarized in Table 2.

Based on coating morphology, coverage, and integrity, the optimal deposition variant was selected for subsequent Zn incorporation.

### 2.5. Preparation of Zn-Containing CaP Coatings

Zn-containing CaP coatings were prepared following the same deposition procedure described above. Zinc chloride (ZnCl_2_, supplied by Sigma-Aldrich, St. Louis, MO, USA) was used as the Zn^2+^ source and was added 3 min after the addition of the last salt. Three Zn^2+^ concentrations were investigated to evaluate the effect of Zn content on coating chemistry, cell viability, antibacterial activity, and Zn^2+^ release behavior. The ZnCl_2_ masses and corresponding Zn^2+^ concentrations are summarized in Table 3.

The Zn^2+^ concentrations employed in this study were selected based on a combination of literature evidence and preliminary screening experiments. Specifically, the chosen range was designed to: (i) remain within biologically relevant limits, where Zn^2+^ is known to promote osteogenic activity without inducing significant cytotoxic effects. Previous studies indicate that Zn concentrations in the low ppm range can enhance osteoblast function while maintaining acceptable cell viability, (ii) enable differentiation between incorporation regimes, from low Zn content (favoring surface-adsorbed species) to higher concentrations (increasing the likelihood of structural incorporation into the CaP lattice). This was essential to evaluate how the physicochemical state of Zn influences release kinetics and biological response, (iii) avoid excessive supersaturation or uncontrolled precipitation in solution, which could lead to uncontrolled phase formation or compositional inhomogeneity during biomimetic deposition.

Additionally, preliminary trials were conducted to identify concentration thresholds that produce measurable antibacterial effects without compromising coating integrity or cytocompatibility. These preliminary tests conducted in this study indicated that Zn concentrations above 2.0 mmol/L led to unstable coatings with reduced structural integrity and non-uniform morphology. Therefore, 2.0 mmol/L was selected as a practical upper limit that ensures a balance between effective Zn incorporation, coating stability, and biological compatibility.

The selected conditions (VDZn1, VDZn2, VDZn3) therefore represent a controlled compositional gradient that directly supports the central hypothesis regarding the relationship between Zn incorporation, release behavior, and multifunctional performance.

### 2.6. Post-Deposition Thermal Treatment

After coating deposition, all samples were subjected to a post-deposition thermal treatment in a muffle furnace. The heating protocol consisted of the following steps: (i) heating to 500 °C and holding for 30 min, (ii) increasing temperature to 800 °C and holding for 1 h, and (iii) cooling inside the furnace to room temperature. This thermal treatment was applied to improve coating cohesion, interparticle bonding, and coating–substrate adhesion.

### 2.7. Surface and Chemical Characterization

#### 2.7.1. Field Emission Scanning Electron Microscopy (FESEM)

Surface morphology and topography were examined using a field emission scanning electron microscope (FESEM, SU8000, Hitachi High-Technologies Corporation, Tokyo, Japan). Images were acquired using an accelerating voltage of 2 kV and an in-lens secondary electron detector.

The thickness of the coatings was determined through cross-sectional analysis using field emission scanning electron microscopy (FESEM). Representative cross-sectional micrographs were obtained by mechanically grinding the edge of the coated Ti6Al4V specimens to expose the coating–substrate interface, allowing its direct observation. Multiple measurements were taken at different locations along the cross-section to account for local variations and ensure statistical reliability.

#### 2.7.2. Energy Dispersive X-Ray Spectroscopy (EDS)

Elemental surface composition was analyzed by energy dispersive X-ray spectroscopy (EDS) scanning electron microscope (JSM-6510LV, JEOL Ltd., Tokyo, Japan) equipped with an EDS detector. Analyses were performed at an accelerating voltage of 20 kV to obtain semi-quantitative elemental information.

#### 2.7.3. X-Ray Photoelectron Spectroscopy (XPS)

Surface elemental composition and chemical states were analyzed by X-ray photoelectron spectroscopy (XPS, K-Alpha™, Thermo Fisher Scientific, East Grinstead, UK) using Al Kα radiation (hν = 1486.68 eV). Binding energies were calibrated using the C1s peak at 284.8 eV to correct for possible charging effects, ensuring accurate binding energy assignment. Survey spectra and high-resolution spectra for P, Zn, Na, Ca, O, and C were collected for all samples. The XPS data were processed using appropriate background subtraction (Shirley-type) and peak deconvolution procedures with well-established fitting parameters, including consistent full width at half maximum (FWHM) and Gaussian–Lorentzian peak shapes. Depth profiling was performed by Ar^+^ ion sputtering at a rate of 1.19 nm/s for 30 s, followed by repeated spectral acquisition. Data processing was performed using Thermo Scientific™ Avantage™ software (version 5.9925, Thermo Fisher Scientific, East Grinstead, UK).

#### 2.7.4. X-Ray Diffraction (XRD)

Crystalline phases were identified by X-ray diffraction (XRD, Empyrean, Malvern Panalytical Ltd., Malvern, UK). Patterns were recorded over a 2θ range of 10–90°, operating at 45 kV and 40 mA, with a scanning speed of 0.013° per 10 s. Cu Kα radiation was used as the X-ray source.

#### 2.7.5. Surface Roughness

Surface roughness was measured using a contact profilometer (SJ-210, model 178-563-01A, Mitutoyo Corporation, Kawasaki, Japan). Measurements were performed on both treated and coated surfaces to assess the effect of surface modification and coating deposition.

The surface roughness parameters Ra, Rz, and Rq were evaluated. Ra represents the average absolute deviation of the surface profile from the mean line and provides a general indication of overall surface texture. Rq is the square root of the mean of the squared deviations, giving greater weight to larger surface irregularities and thus being more sensitive to outliers than Ra. Rz (maximum height of the profile) describes the vertical distance between the highest peak and the deepest valley within the evaluation length, providing information about extreme surface features and peak-to-valley variations. Together, these parameters allow a more comprehensive characterization of surface topography changes induced by the treatments.

### 2.8. In Vitro Biological Characterization

#### 2.8.1. Cytotoxicity Assay (MTT)

Cytotoxicity was evaluated using the MTT reduction assay according to ISO 10993-5:2009, Annex C (*Biological Evaluation of Medical Devices—Part 5: Tests for In Vitro Cytotoxicity*; International Organization for Standardization (ISO): Geneva, Switzerland, 2009). Primary human gingival fibroblasts (HGF, ATCC^®^ PCS-201-108™) and human dermal fibroblasts (HDF, ATCC^®^ PCS-2018-012) were used. Cells were cultured in Dulbecco’s Modified Eagle Medium (DMEM) supplemented with 10% fetal bovine serum (FBS), 100 U/mL penicillin, and 100 μg/mL streptomycin. Cell suspensions were prepared at concentrations of approximately 4.0 × 10^4^ cells/mL (HGF) and 5.0 × 10^4^ cells/mL (HDF). One milliliter of cell suspension was seeded per well and incubated for 24 h at 37 °C in a humidified atmosphere with 5% CO_2_ to allow cell attachment. After incubation, the test materials were placed in the wells along with negative controls (culture medium) and positive controls (5% Triton X-100). After 24 h of exposure, samples and culture medium were removed. Fresh medium (200 μL) and MTT solution (60 μL) were added to each well, and plates were incubated for 4 h in the dark. The resulting formazan crystals were dissolved using 200 μL of dimethyl sulfoxide (DMSO) per well. Absorbance was measured at 570 nm using a microplate reader (Thermo Scientific MultiSkan GO, Thermo Fisher Scientific Inc., Waltham, MA, USA).

Cell viability was calculated as:

(1) $$Cell\;viability\;\left(\%\right)=\frac{OD\;of\;treated\;cells}{OD\;of\;control\;cells}\times 100$$

where *OD* = optical density.

#### 2.8.2. Antibacterial Activity Assay

Antibacterial activity was evaluated against the Gram-positive bacterium *Streptococcus mutans*. Bacterial cultures were grown in Brain Heart Infusion (BHI) medium at 37 °C for 24 h. The bacterial concentration was determined using a Neubauer counting chamber and adjusted to 1 × 10^6^ CFU/mL. Each coated specimen was incubated in tubes containing 200 μL of bacterial suspension for 24 h. After incubation, samples were removed and rinsed with phosphate-buffered saline (PBS) to remove loosely attached bacteria. Serial dilutions (1:10) were prepared, and 100 μL from the final dilution was plated onto nutrient agar plates. Plates were incubated for 24 h at 37 °C prior to colony counting.

Colonies were counted, and *CFU/mL* was calculated as:

(2) $$CFU/mL=\frac{\left(number\;of\;colonies\times dilution\;factor\right)}{sample\;volume\;\left(mL\right)}$$

The percentage reduction in bacterial colonies relative to the undoped CaP control was calculated.

(3) $$Percentaje\;reduction\;\left(\%\right)=100-\left[\frac{\left(CFU\;of\;the\;control\;surface\right)}{\left(CFU\;of\;the\;coated\;surface\right)}\times 100\right]$$

### 2.9. Zn^2+^ Ion Release Test

Zn^2+^ release from Zn-containing coatings was evaluated in phosphate-buffered saline (PBS, 1×, pH 7.4). Samples were immersed in 50 mL of PBS at room temperature. The solution was replaced with fresh PBS, and Zn^2+^ concentrations were measured after 1, 3, and 7 days. Zn^2+^ concentrations were determined by atomic absorption spectroscopy (AAS, iCE™ 3000, Thermo Fisher Scientific, Cambridge, UK). Measurements were performed at an emission wavelength of 213.9 nm, with a detection limit of 0.002 μg/mL. Results were reported as μg/mL of Zn^2+^ released into the solution.

### 2.10. Numbers of Repetition, Statistical Analysis, and Experimental Reproducibility

All experiments were conducted in triplicate (*n* = 3) unless otherwise specified. This includes: surface characterization (SEM/EDS, XRD, XPS, roughness), ion release measurements (AAS), antibacterial assays (CFU counting), and cytotoxicity tests.

For each condition, independent samples were prepared and analyzed to account for sample-to-sample variability. The reproducibility of the biomimetic coatings was evaluated through consistent morphological features, phase composition, and Zn incorporation trends across replicates.

We observed low variability between samples, with standard deviations generally below 10% for quantitative measurements such as Zn release and biological assays, indicating good reproducibility of the deposition process.

The experimental data were analyzed using standard statistical methods to ensure reliability and significance: results are reported as means ± standard deviation (SD). Statistical comparisons between groups were performed using one-way analysis of variance (ANOVA). When significant differences were detected, post hoc Tukey tests were applied. A significance level of *p* < 0.05 was considered statistically significant.

The reproducibility of the coatings was confirmed through: (i) consistent surface morphology observed by SEM across independent samples, (ii) stable phase composition verified by XRD patterns showing reproducible apatite-related peaks, (iii) comparable Zn incorporation trends confirmed by EDS and XPS analyses, and (iv) reproducible ion release profiles with similar kinetic behavior across replicates.

The biomimetic deposition method, combined with controlled surface activation and solution chemistry, contributed to a high level of process stability. Minor variations observed are attributed to the intrinsic heterogeneity of nucleation processes in biomimetic systems, which is a known characteristic of this technique.

## 3. Results

### 3.1. Baseline Characterization of the Ti6Al4V Substrate

**Figure 1:**
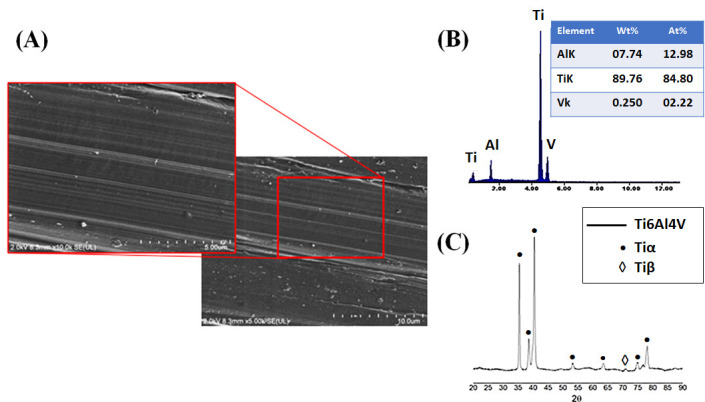
Ti6Al4V ELI alloy machined surface prior to surface modification. (**A**) FESEM images, 5000× and 10,000×. (**B**) EDS spectrum. (**C**) XRD pattern.

Prior to surface modification, the Ti6Al4V ELI alloy was characterized to establish a reference microstructural, compositional, and crystallographic state for assessing the effects of subsequent chemical and thermal treatments (Figure 1A–C). FESEM images in Figure 1A revealed a typical machined surface morphology dominated by parallel grooves and tool marks, resulting in an anisotropic topography. Such as-machined surfaces are well known to exhibit low surface energy, a limited density of chemically active sites, and reduced wettability, which collectively limit heterogeneous nucleation of calcium phosphate phases [55]. From a biological and bioactivity perspective, this surface condition is considered essentially bioinert and exhibits a limited intrinsic ability to induce apatite formation in simulated body fluid (SBF). This behavior is consistent with extensive reports showing that untreated Ti6Al4V surfaces display poor apatite-forming ability compared to chemically activated, hydroxylated, or microtextured surfaces [56].

The EDS analysis in Figure 1B confirmed the expected elemental composition of the alloy, with dominant Ti, Al, and V signals and no detectable contamination. Meanwhile, the XRD patterns in Figure 1C revealed the coexistence of α-Ti (hcp) and β-Ti (bcc) phases, confirming the typical biphasic microstructure of Ti6Al4V used in biomedical applications. The baseline characterization confirms that the as-machined substrate provides a chemically passive and weakly bioactive surface, which requires surface activation to enable effective biomimetic coating nucleation and growth [57].

### 3.2. Effect of Surface Activation Treatments

#### 3.2.1. Acid Etching

**Figure 2:**
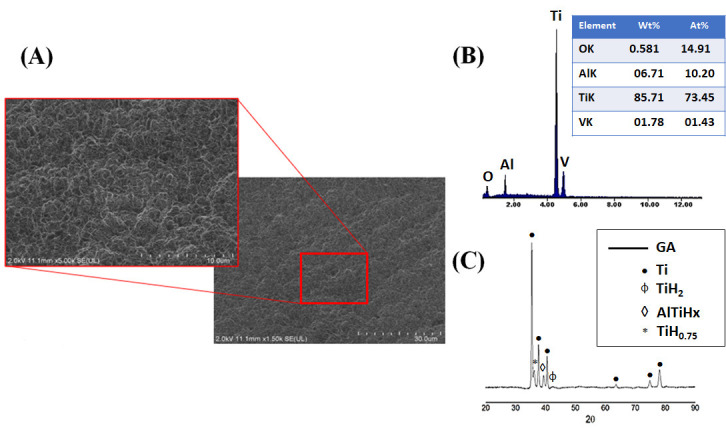
Ti6Al4V ELI alloy after acid etching. (**A**) FESEM images, 1500× and 5000×. (**B**) EDS spectrum. (**C**) XRD pattern.

Figure 2A–C shows the microstructural, compositional, and crystallographic state of Ti6Al4V ELI alloy after acid etching. According to the FESEM images in Figure 2A, acid etching using an H_2_SO_4_/HCl mixture produced a marked transformation of the surface topography. The machining marks were effectively removed and replaced by a homogeneous network of micropores. This microtextured morphology originates from preferential chemical dissolution at high-energy regions such as grain boundaries, phase interfaces, and dislocation-rich areas, leading to a substantial increase in surface roughness, specific surface area, and the density of chemically active sites [58].

Such microporosity is known to enhance both mechanical interlocking and heterogeneous nucleation of calcium phosphate phases by increasing the number of energetically favorable nucleation sites. Importantly, no significant microcracks were observed, indicating that the selected etching conditions achieved effective surface activation without inducing detrimental structural damage, in agreement with previous reports on acid-treated titanium alloys [59]. The EDS analysis in Figure 2B revealed an increase in oxygen content associated with the spontaneous formation of a thin titanium oxide layer upon exposure to air. Finally, the XRD analysis in Figure 2C detected the presence of titanium hydride (TiH_2_), which can form during strong acid treatments due to hydrogen uptake. The formation of TiH_2_ has been reported to modify surface chemistry and surface energy and can influence subsequent alkaline treatments by altering dissolution and reprecipitation kinetics [60].

#### 3.2.2. Alkaline and Calcium Activation (NaOH–CaCl_2_–TT–H_2_O)

**Figure 3:**
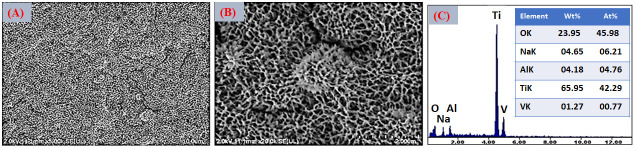
Ti6Al4V ELI alloy after alkaline treatment in NaOH. (**A**) FESEM image, 5000×. (**B**) FESEM image, 20,000×. (**C**) EDS spectrum.

**Figure 4:**
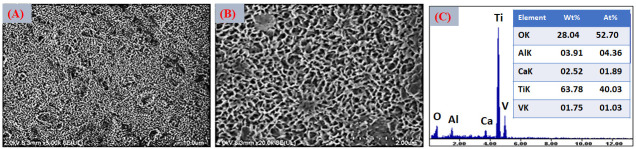
Ti6Al4V ELI alloy after CaCl_2_ treatment. (**A**) FESEM image, 5000×. (**B**) FESEM image, 20,000×. (**C**) EDS spectrum.

**Figure 5:**
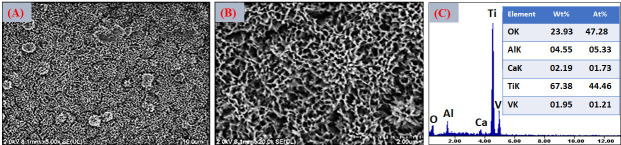
Ti6Al4V ELI alloy after thermal treatment, 600 °C for 1h. (**A**) FESEM image, 5000×. (**B**) FESEM image, 20,000×. (**C**) EDS spectrum.

The morphological evolution of the Ti6Al4V ELI surface after the sequential application of surface activation treatments is shown in Figure 3, Figure 4 and Figure 5. Following alkaline treatment in NaOH, FESEM images in Figure 3A–B revealed the formation of a highly porous, fibrillar, or feather-like network structure, characteristic of hydrated sodium titanate layers. This morphology results from partial dissolution of the native TiO_2_ layer followed by reprecipitation of sodium titanate species, leading to the formation of a gel-like network enriched in Ti–OH groups. Such titanate gel layers are widely recognized as key intermediates for bioactivation, as the high density of surface hydroxyl groups provides negatively charged sites that strongly promote Ca^2+^ adsorption and subsequent apatite nucleation [61]. EDS analysis performed after NaOH treatment (Figure 3C) confirmed the substantial incorporation of sodium and oxygen, in addition to the substrate elements Ti, Al, and V. The simultaneous detection of Na and O supports the formation of a sodium titanate layer (commonly described as Na_2_TiO_3_ or structurally related sodium titanate species), which has been consistently identified as an essential phase responsible for inducing bioactivity on titanium surfaces. Approximately 6 at.% Na was detected at the outer surface, indicating the effective development of a sodium-rich hydrated titanate layer. This compositional modification is consistent with the fibrillar gel-like morphology observed by FESEM and reflects the partial dissolution–reprecipitation mechanism induced under alkaline conditions. The resulting titanate network behaves as an ion-exchangeable reservoir, where loosely bound Na^+^ ions can be replaced by H_3_O^+^ upon subsequent immersion in aqueous environments. This exchange process increases the density of surface Ti–OH groups and enhances the negative surface charge, thereby strengthening electrostatic interactions with Ca^2+^ ions from the surrounding medium. These physicochemical features are critical for initiating calcium accumulation and, ultimately, for driving heterogeneous apatite nucleation during subsequent biomimetic mineralization.

After CaCl_2_ treatment, the fibrillar morphology was preserved, as shown in the FESEM images in Figure 4A,B, while globular precipitates appeared, indicating the formation of calcium-rich titanate species. The EDS analysis in Figure 4C confirmed the disappearance of Na and the appearance of Ca, demonstrating an effective Na^+^/Ca^2+^ ion exchange. This ion-exchange process leads to the formation of calcium titanate or calcium hydrogen titanate layers, which are recognized as highly effective precursors for rapid apatite formation in SBF [62].

FESEM images in Figure 5A,B revealed that thermal treatment induced partial densification and the formation of microcracks, attributed to dehydration and shrinkage of the gel-derived layer. Although microcracking may appear undesirable, such features can enhance ionic permeability and facilitate ion exchange with the surrounding medium, thereby accelerating interfacial reactions and apatite nucleation kinetics [63].

Subsequent thermal treatment did not produce significant changes in the elemental composition detected by EDS, indicating that calcium remained stably incorporated within the surface layer. The persistence of Ca after heat treatment suggests that calcium species were not merely adsorbed but were structurally integrated into the titanate matrix, likely through the formation of calcium titanate–type phases or Ca-associated titanate domains [64]. This compositional stability is consistent with the partial densification observed in FESEM images and implies that thermal consolidation promoted structural rearrangement rather than elemental loss. During heating, dehydration of the gel-derived sodium/calcium titanate layer facilitates condensation reactions between Ti–OH groups, leading to a more interconnected oxide network while preserving incorporated Ca species. The retention of calcium is particularly relevant, as Ca-enriched titanate surfaces exhibit enhanced chemical affinity for phosphate ions in physiological environments [65]. In combination with the microcrack network generated during shrinkage, this stable Ca-containing layer can further promote ionic transport and accelerate the initial stages of calcium phosphate nucleation during subsequent biomimetic immersion.

Treatment in deionized water (TH_2_O) did not lead to noticeable morphological changes, which is consistent with its primary role in hydrating and chemically stabilizing the surface layer rather than altering its topography. This step mainly promotes further ion exchange and the formation of additional surface hydroxyl groups, contributing to the chemical conditioning of the titanate layer without significantly affecting its microstructural features.

**Figure 6:**
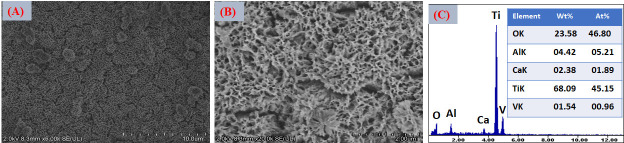
Ti6Al4V ELI alloy after deionized water treatment. (**A**) FESEM image, 5000×. (**B**) FESEM image, 20,000×. (**C**) EDS spectrum.

A slight reduction in Ca content after the deionized water treatment (Figure 6) was detected by EDS. This decrease can be attributed to the partial dissolution of weakly bound calcium species, likely those superficially adsorbed or associated with more labile domains of the hydrated titanate layer [66]. Importantly, this reduction did not compromise the overall presence of calcium at the surface, indicating that a significant fraction remained structurally integrated within the titanate network.

Such behavior is consistent with the role of the TH_2_O step as a chemical conditioning stage rather than a structural modification process. Immersion in deionized water promotes further Na^+^/H_3_O^+^ exchange and stabilization of Ti–OH groups, while simultaneously removing loosely attached ionic species. The retention of a Ca-enriched surface after this mild leaching step suggests that calcium is at least partially incorporated through stronger chemical interactions, which may enhance the surface’s readiness for subsequent phosphate adsorption and calcium phosphate nucleation under biomimetic conditions [67].

**Figure 7:**
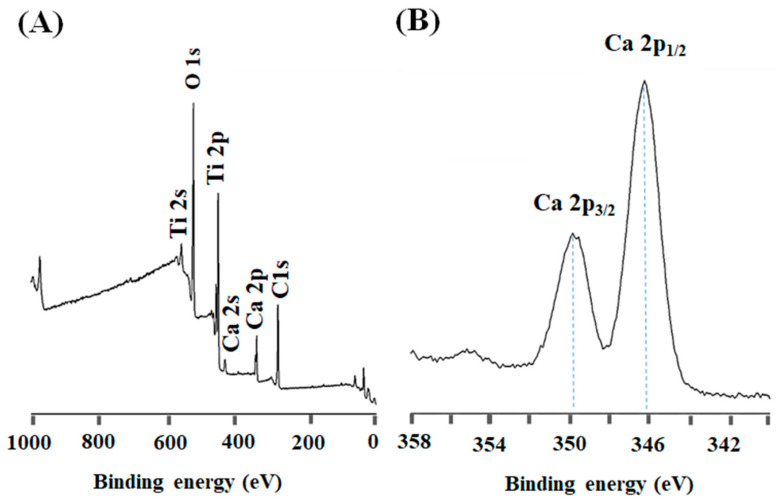
(**A**) Full XPS spectrum and (**B**) High-resolution Ca 2p spectrum of the Ti6Al4V surface subjected to NaOH-CaCl_2_-TT-TH_2_O activation treatments.

Figure 7A,B presents the XPS characterization of the Ti6Al4V ELI surface after the full sequence of activation treatments (NaOH–CaCl_2_–TT–TH_2_O). The survey spectrum (Figure 7A) displays well-defined signals corresponding to Ti, O, and Ca. The absence of intense metallic Ti signals and the predominance of oxidized species indicate that the substrate is fully covered by a reaction layer formed during the alkaline and calcium treatments. High-resolution analysis of the Ca 2p region (Figure 7B) reveals the characteristic Ca 2p_3_/_2_ and Ca 2p_1_/_2_ doublet, consistent with Ca^2+^ chemical states.

The two peaks in the Ca 2p region with binding energies of 346.8 eV and 350.3 eV are in excellent agreement with the characteristic positions of Ca 2p_1/2_ and Ca 2p_3/2_, respectively. The binding energy positions and peak symmetry suggest that calcium is not present as a simple surface precipitate but is chemically associated with oxygen within a titanate-based matrix, likely through Ca–O–Ti linkages or calcium titanate–like environments. This interpretation supports the EDS findings and indicates that Ca is stably incorporated within the outer nanometric layer rather than weakly adsorbed. Importantly, XPS (being inherently surface-sensitive) confirms that calcium enrichment occurs precisely at the interface where biomimetic reactions are initiated [68].

**Figure 8:**
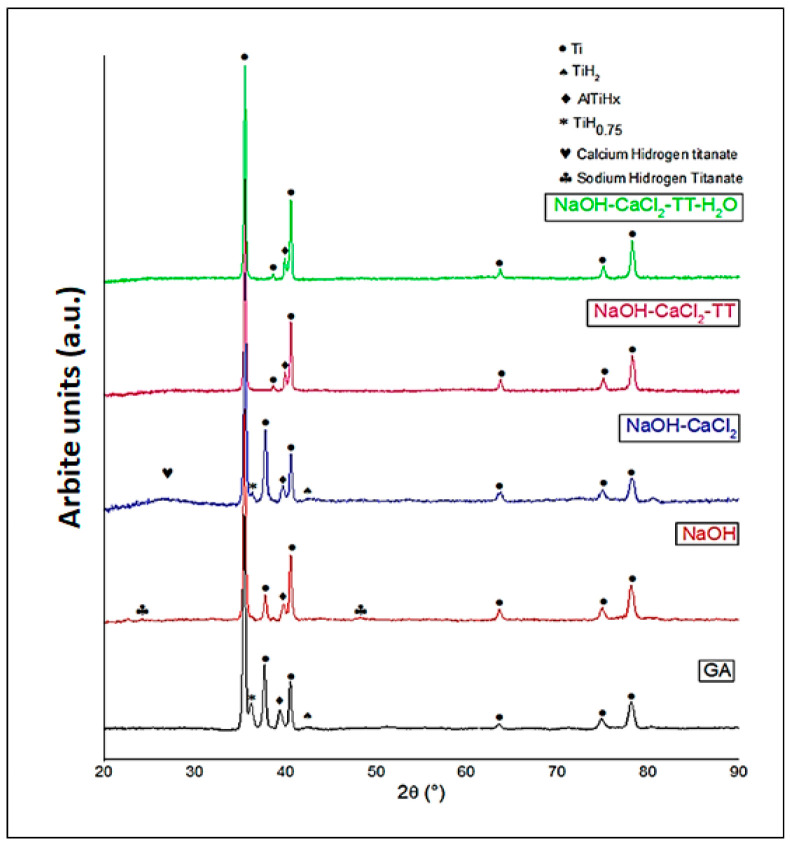
XRD patterns of the surfaces after the application of the activation treatments.

XRD analysis (Figure 8) provides detailed insight into the phase evolution occurring at each stage of the activation sequence applied to Ti6Al4V. In the acid-etched condition, the diffraction pattern was dominated by α-Ti reflections from the substrate, together with peaks indexed to TiH_0.75_, AlTiHx, and calcium hydrogen titanate. The formation of titanium hydrides is commonly associated with hydrogen uptake during acid exposure, where cathodic reactions at the metal surface promote hydrogen absorption into the near-surface region. Although typically confined to shallow depths, these hydride phases can introduce lattice distortion and defect sites, potentially influencing subsequent ion exchange and chemical reactivity.

After NaOH treatment, reflections corresponding to Ti and AlTiHx remained, while sodium hydrogen titanate phases became detectable. The emergence of sodium titanate confirms the dissolution–reprecipitation mechanism previously inferred from FESEM, EDS, and XPS analyses. Under strongly alkaline conditions, partial dissolution of the native TiO_2_ layer is followed by reprecipitation of a poorly crystalline sodium titanate hydrogel. The weak and broadened nature of these reflections indicates small crystallite size and significant structural disorder, consistent with a gel-derived surface layer. For the NaOH–CaCl_2_ sequence, calcium hydrogen titanate reflections were identified along with TiH_0.75_, Ti, AlTiHx, and TiH_2_. This phase assemblage evidences partial Na^+^/Ca^2+^ exchange within the titanate network, resulting in the formation of Ca-containing titanate species. The persistence of hydrides suggests that calcium treatment primarily modifies the outer titanate structure without fully eliminating hydrogen-enriched domains. Following thermal treatment (NaOH–CaCl_2_–TT), only Ti and AlTiHx peaks were clearly observed. According to the literature, heat treatment can transform hydrogen and calcium titanates into low-crystallinity calcium titanate phases and rutile. However, such phases were not distinctly identified in the present study. This apparent discrepancy can be rationalized by considering that (i) the activated layer is extremely thin, (ii) it exhibits low crystallinity or partial amorphous character, and (iii) its diffraction signal is masked by the intense contribution of the metallic substrate. Similar limitations of XRD for detecting nanometric calcium titanate layers have been widely reported, with surface-sensitive techniques such as XPS and EDS demonstrating greater sensitivity for these modified interfaces. The final NaOH–CaCl_2_–TT–TH_2_O condition exhibited a comparable diffraction pattern, further supporting the conclusion that the outer layer is nanometric and structurally disordered. Collectively, the progressive attenuation of titanate-related peaks confirms the formation of a thin, calcium-enriched, highly hydroxylated, and chemically reactive surface. This activated interface provides an optimal physicochemical platform for subsequent biomimetic calcium phosphate deposition and controlled Zn^2+^ incorporation, enhancing nucleation kinetics, coating adhesion, and interfacial stability. These characteristics are central to achieving both bioactivity and antibacterial functionality, in line with the core hypothesis of the study [69].

### 3.3. Biomimetic Deposition of CaP Coatings

The influence of ionic concentration (SBFX7 vs. SBFX10) and immersion time (4 h vs. 6 h) on CaP deposition was systematically evaluated. The results clearly demonstrate that both parameters strongly control coating morphology, surface coverage, thickness, and mechanical integrity, in agreement with classical biomimetic deposition models [70].

**Figure 9:**
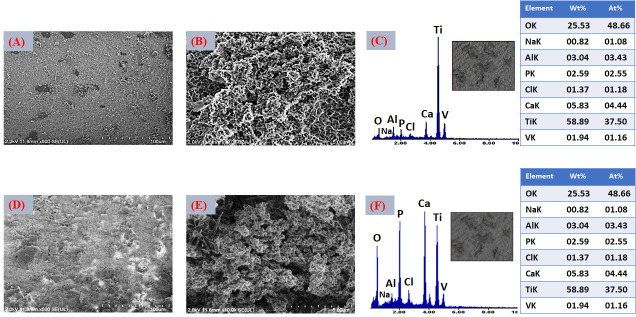
(**A**,**B**) FESEM images of the coated surface with VD1 (SBFX7- 4 h), 500× and 10,000×. (**C**) EDS spectra of the surfaces coated with VD1. (**D**,**E**) FESEM images of the thermally treated coated surface with VD1, 500× and 10,000×. (**F**) EDS spectra of the surfaces coated with thermally treated VD1.

FESEM micrographs in Figure 9A,B corresponding to the VD1 condition (SBFX7) reveal the formation of spherical particles arranged in bouquet-like clusters, heterogeneously distributed across the substrate surface.

This morphology is typical of the early stages of biomimetic apatite nucleation, where nanoscale crystallites initially aggregate into globular assemblies that progressively coalesce into more continuous layers. Such features indicate that nucleation is occurring at discrete active sites rather than uniformly over the entire surface, reflecting local variations in interfacial chemistry [71].

The presence of larger lamellar structures underlying or supporting the bouquet-like aggregates suggests the coexistence of distinct growth mechanisms. These lamellae may originate from preferential nucleation in regions with higher surface hydroxyl density or locally increased calcium concentration within the activated titanate layer. Surfaces enriched in calcium titanate are known to promote heterogeneous nucleation by facilitating electrostatic interactions with phosphate species, which can lead to localized crystal growth and morphological differentiation. Elemental composition was evaluated by EDS. The average atomic percentages (Figure 9C) confirmed the presence of Ca (4.44 at.%), P (2.55 at.%), and O (48.66 at.%), consistent with the formation of a thin calcium phosphate layer.

After thermal treatment (Figure 9D,E), the coating appears more compact, with no evident cracking. This behavior indicates that the relatively low thickness of the deposited layer allows accommodation of thermal stresses generated during heating [72].

Nevertheless, the coating remains partially discontinuous, suggesting that the immersion time (4 h) and the ionic concentration of the supersaturated solution (SBFX7) were insufficient to achieve full surface coverage. From a functional perspective, incomplete homogeneity may affect coating adhesion, bioactivity reproducibility, and the subsequent uniform incorporation of Zn^2+^. Localized analyses performed on the lamellar regions (Figure 9F) showed significantly higher Ca and P contents (11.25 and 8.87 at.%, respectively; O: 62.95 at.%), confirming that these structures correspond to CaP-rich domains. The increase in Ca and P after heat treatment suggests slight densification and partial structural rearrangement, without fundamentally altering the coating chemistry. The compositional and morphological evidence supports the formation of a thin, apatite-like calcium phosphate layer on the Ti6Al4V substrate.

**Figure 10:**
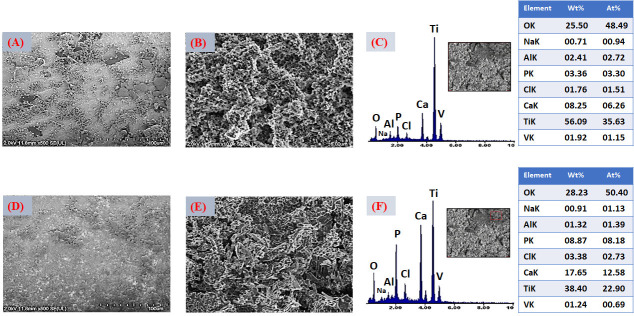
(**A**,**B**) FESEM images of the coated surface with VD2 (SBFX7- 6 h), 500× and 10,000×. (**C**) EDS spectra of the surfaces coated with VD2. (**D**,**E**) FESEM images of the thermally treated coated surface with VD2, 500× and 10,000×. (**F**) EDS spectra of the surfaces coated with thermally treated VD2.

For the VD2 condition (Figure 10A,B), increasing the immersion time to 6 h while maintaining the same ionic concentration (SBFX7) resulted in a higher density of bouquet-like aggregates and the formation of more compact clusters distributed over a larger fraction of the surface. This morphology reflects further progression of secondary nucleation and crystal growth, leading to increased surface coverage compared with VD1. As immersion proceeds, previously formed nuclei act as templates for additional ion accumulation, promoting radial growth and coalescence of adjacent globular structures. Despite this improvement, lamellar features and partially uncovered regions are still evident, indicating that although extended time enhances growth kinetics, the ionic supersaturation remains a limiting factor for achieving a fully continuous layer. In other words, time alone promotes thickening and local densification but does not completely overcome constraints imposed by ion availability and diffusion [73]. EDS analysis (Figure 10C) showed increased atomic concentrations of Ca (6.26 at.%) and P (3.30 at.%) relative to VD1, confirming that longer immersion favors greater CaP accumulation. After thermal treatment (Figure 10D,E), the coating exhibits further densification without visible cracking, suggesting that the layer thickness remains within a range capable of accommodating thermally induced stresses. Heat treatment likely promotes partial dehydration and structural rearrangement of the initially precipitated calcium phosphate, improving interparticle cohesion without causing delamination.

EDS analysis (Figure 10F) showed increased atomic concentrations of Ca and P relative to VD1, confirming that longer immersion favors greater CaP accumulation. Additionally, EDS analysis (Figure 10F) indicates that the increase in Ca and P content after heat treatment is consistent with slight densification and partial structural rearrangement of the coating. Significantly higher Ca and P atomic concentrations (12.58 and 8.18 at.%, respectively; O: 50.4 at.%) were measured in these regions, confirming that they correspond to CaP-rich domains. These results further support that longer immersion times promote greater CaP accumulation and local thickening of the deposited layer.

From a process optimization standpoint, these findings indicate that extending deposition time without increasing ionic supersaturation only partially improves surface coverage. Achieving a continuous and homogeneous layer (critical for reliable bioactivity and subsequent Zn functionalization) likely requires adjustments to both immersion time and solution chemistry.

**Figure 11:**
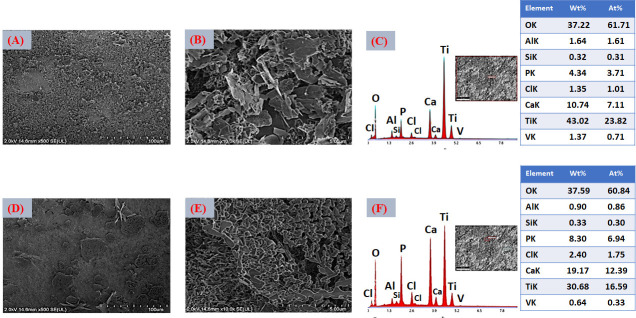
(**A**,**B**) FESEM images of the coated surface with VD3 (SBFX10- 4 h), 500× and 10,000×. (**C**) EDS spectra of the surfaces coated with VD3. (**D**,**E**) FESEM images of the thermally treated coated surface with VD3, 500× and 10,000×. (**F**) EDS spectra of the surfaces coated with thermally treated VD3.

FESEM micrographs corresponding to the VD3 condition (Figure 11A,B) show a clear improvement in coating uniformity and continuity compared with VD1 and VD2. Increasing the Ca^2+^ and PO_4_^3−^ concentrations in the solution (SBFX10) enhances the degree of supersaturation, which in turn raises the heterogeneous nucleation rate and promotes the formation of a thicker and more homogeneous layer, even at a relatively short immersion time (4 h). Under these conditions, the higher ionic activity reduces the energy barrier for nucleation and increases the density of stable nuclei formed on the activated titanate surface. This behavior is consistent with classical biomimetic deposition models, where supersaturation governs both nucleus density and crystal growth kinetics. A higher supersaturation level accelerates ion clustering at reactive surface sites, leading to more uniform spatial coverage and faster lateral growth of calcium phosphate domains [74]. EDS analysis (Figure 11C) revealed increased Ca (7.11 at.%) and P (3.71 at.%) contents compared with the previous variants, confirming greater CaP deposition. After thermal treatment (Figure 11D,E), the layer exhibits more pronounced densification, with visible “necks” forming between adjacent particles. This feature is indicative of solid-state sintering or partial crystal coalescence, suggesting improved interparticle bonding. Such microstructural evolution can enhance the internal cohesion of the coating and strengthen its adhesion to the substrate, which are crucial factors for mechanical stability under physiological loading.

EDS analysis (Figure 11F) revealed increased Ca (12.39 at.%) and P (6.94 at.%) contents, confirming greater CaP deposition, which can promote possible continuous CaP layer formation. From a coating design perspective, VD3 represents a favorable balance between thickness, uniformity, and crack-free morphology. These characteristics are particularly advantageous for subsequent Zn^2+^ incorporation and for achieving controlled, reproducible ionic release in multifunctional implant applications.

**Figure 12:**
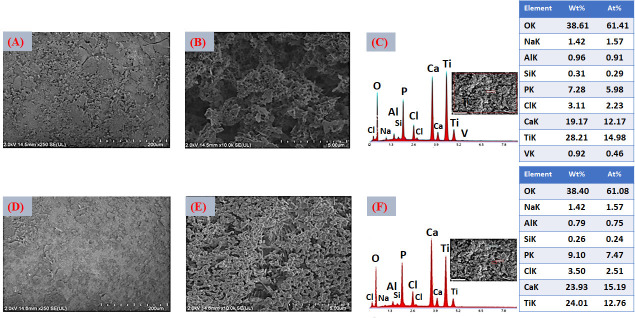
(**A**,**B**) FESEM images of the coated surface with VD4 (SBFX10- 6 h), 500× and 10,000×. (**C**) EDS spectra of the surfaces coated with VD4. (**D**,**E**) FESEM images of the thermally treated coated surface with VD4, 500× and 10,000×. (**F**) EDS spectra of the surfaces coated with thermally treated VD4.

The VD4 condition (Figure 12A,B), which combines higher ionic concentration (SBFX10) with extended immersion time (6 h), produced the thickest and most compact layer among all the variants evaluated. The surface exhibits nearly complete coverage, with densely packed particle agglomerates indicative of sustained secondary nucleation and continuous crystal growth. Under these highly supersaturated conditions, both the nucleation density and the growth rate are maximized, resulting in substantial accumulation of calcium phosphate across the activated substrate [75]. However, localized cracking is evident in certain regions. This behavior suggests that excessive layer thickness generates residual stresses, likely arising from mismatches in thermal expansion coefficients between the CaP coating and the Ti6Al4V substrate, as well as shrinkage during drying and subsequent heat treatment. EDS analysis (Figure 12C) revealed the highest Ca (12.17 at.%) and P (5.98 at.%) concentrations among all variants, confirming that VD4 yields the greatest CaP deposition. After thermal treatment (Figure 12D,E), cracking becomes more pronounced, although the formation of interparticle “necks”, similar to those observed in VD3, indicates partial sintering and improved local cohesion. While densification enhances particle bonding, excessive thickness compromises the overall mechanical integrity of the coating. The post-heat-treatment EDS (Figure 12F) confirmed increased Ca (15.19 at.%) and P (7.47 at.%) contents, confirming greater CaP deposition that might promote a CaP continuous layer homogeneously distributed with full surface coverage.

Despite maximizing CaP loading, the presence of cracks represents a critical limitation of the VD4 variant. Such defects may promote coating delamination under mechanical stress and lead to uncontrolled ionic release [76]. Therefore, although VD4 achieves the highest deposition efficiency, it does not necessarily represent the optimal balance between thickness, structural integrity, and functional reliability required for biomedical applications.

A comparative assessment of the four deposition variants clearly demonstrates that both ionic concentration and immersion time play a decisive role in controlling coating morphology, uniformity, thickness, and structural stability. VD1 and VD2 resulted in relatively thin and heterogeneous layers with incomplete surface coverage, indicating that limited supersaturation and/or insufficient growth time restrict the formation of a fully continuous calcium phosphate network. Although increasing immersion time (VD2) enhanced CaP accumulation compared with VD1, the coatings remained partially discontinuous. In contrast, VD3 (SBFX10, 4 h) produced a continuous and homogeneous coating with extensive surface coverage and no significant cracking. The higher ionic activity increased supersaturation with respect to apatite, promoting rapid heterogeneous nucleation and sustained lateral crystal growth. As a result, a coherent CaP layer developed within a relatively short deposition time. Post-deposition thermal treatment induced partial densification and the formation of interparticle necks, suggesting improved cohesion and potentially stronger adhesion to the substrate [77]. These features are consistent with optimized biomimetic deposition windows reported in the literature [78]. VD4 (SBFX10, 6 h) generated the thickest coatings and the highest Ca and P contents. However, pronounced cracking was observed, attributed to excessive thickness and residual stresses associated with drying shrinkage and thermal expansion mismatch. This behavior reflects the well-known trade-off between maximizing CaP deposition and maintaining mechanical reliability [79].

VD3 emerges as the most suitable condition for subsequent Zn^2+^ incorporation. It offers an optimal balance of high surface coverage, good homogeneity, absence of significant cracking, and sufficient thickness to accommodate Zn without compromising structural integrity. These characteristics are essential to ensure uniform Zn distribution, controlled ion-release kinetics, and reproducible antibacterial performance, in agreement with the central hypothesis of this study.

### 3.4. Zn-Containing CaP Coatings (Zn-CaP)

Three deposition variants were prepared using different zinc concentrations in the solution (low—VDZn1, intermediate—VDZn2, and high—VDZn3) in order to systematically assess the influence of Zn on coating morphology, composition, and homogeneity, as well as its potential impact on subsequent bioactivity and antibacterial performance. By gradually increasing the Zn^2+^ content in the biomimetic medium, it was possible to examine how this trace element modulates nucleation and crystal growth mechanisms within calcium phosphate (CaP) systems.

**Figure 13:**
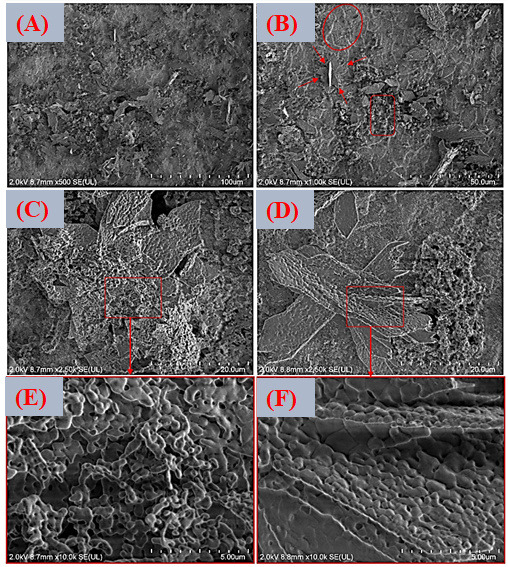
FESEM images of the surface coated with zinc inclusion at the lowest concentration (VDZn1). (**A**) 500×, (**B**) 1000×, (**C**,**D**) 2500×, and (**E**,**F**) 10,000×.

Figure 13A–F shows FESEM micrographs at different magnifications of the surface coated with the lowest zinc concentration (VDZn1) after post-deposition heat treatment. The resulting coating is characterized by clusters of particles forming lamellar assemblies arranged in flower-like configurations. On top of these primary structures, secondary globular precipitates can be observed. This morphology is typical of calcium phosphates produced via biomimetic routes, where heterogeneous nucleation at the substrate–solution interface initially leads to amorphous or poorly crystalline calcium phosphate phases [80]. With time and thermal treatment, these phases tend to reorganize into more ordered apatite-like structures. The radial growth pattern observed in the flower-like structures suggests a diffusion-controlled mechanism combined with anisotropic crystal growth. At low zinc concentrations, the presence of Zn^2+^ does not significantly suppress the formation of these apatite-like assemblies. However, zinc is known to interact with calcium phosphate at the atomic scale, potentially altering surface energy and slightly refining the microstructure. Because Zn^2+^ has a smaller ionic radius than Ca^2+^ and a different electronic configuration, partial substitution within the apatite lattice or adsorption at active growth sites may subtly influence crystal habit and grain size [81].

**Figure 14:**
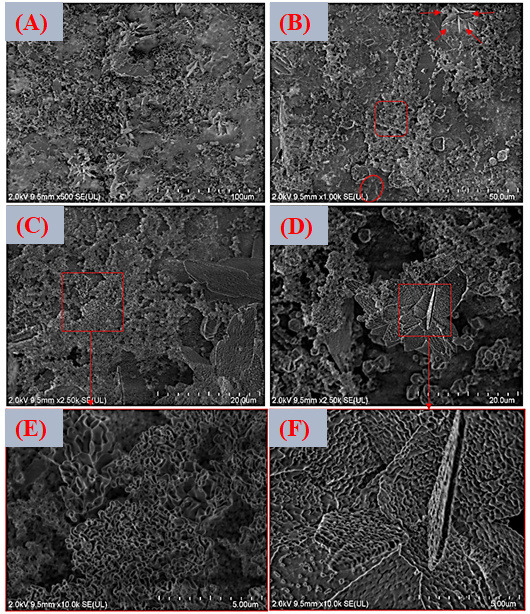
FESEM images of the surface coated with zinc inclusion at the intermediate concentration (VDZn2). (**A**) 500×, (**B**) 1000×, (**C**,**D**) 2500×, and (**E**,**F**) 10,000×.

For the intermediate concentration variant (VDZn2, Figure 14A–F), the morphology remains comparable to that of VDZn1.

Nevertheless, a slight reduction in average particle size and a greater degree of agglomeration fragmentation can be observed. This effect is consistent with the role of Zn^2+^ as a crystallization modifier. Several studies have reported that zinc can partially inhibit apatite crystal growth by competing with Ca^2+^ for lattice positions and inducing local structural distortions. Such distortions may slow down crystal propagation along specific crystallographic directions, resulting in finer and more dispersed particles [82].

**Figure 15:**
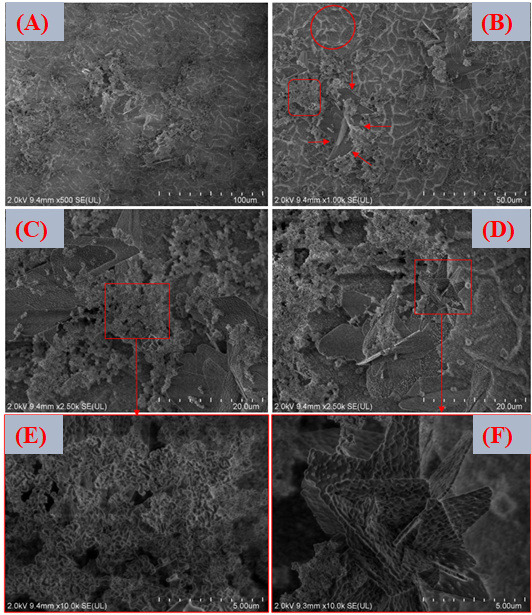
FESEM images of the surface coated with zinc inclusion at the highest concentration (VDZn3). (**A**) 500×, (**B**) 1000×, (**C**,**D**) 2500×, and (**E**,**F**) 10,000×.

In the case of the highest zinc concentration (VDZn3, Figure 15A–F), no drastic qualitative morphological changes are detected when compared with the previous variants. However, a lower surface coverage density and a less homogeneous particle distribution are evident. This behavior suggests that elevated Zn^2+^ concentrations in the solution may limit both nucleation density and subsequent apatite layer growth. Excess zinc can reduce the effective supersaturation with respect to calcium phosphate phases and may stabilize intermediate amorphous species, delaying their transformation into crystalline apatite. As a consequence, the coating appears less continuous and more heterogeneous [83].

The three Zn-containing variants share several microstructural features: (i) non-uniform particle arrangements, (ii) a tendency toward agglomerate formation, (iii) flower-like structures associated with biomimetic apatite, and (iv) increasing surface heterogeneity with higher Zn concentration.

These microstructural characteristics have direct implications for biological performance. Surface roughness, particle size distribution, and heterogeneity strongly influence protein adsorption, early cell adhesion, and subsequent in vivo apatite formation. Moderate roughness and nanoscale features are often beneficial for osteoblastic activity, whereas excessive heterogeneity or poor coverage may compromise mechanical stability and long-term integration [84]. Therefore, controlling zinc concentration is not only a compositional adjustment but also a strategy to tailor surface architecture for optimized biological response.

**Figure 16:**
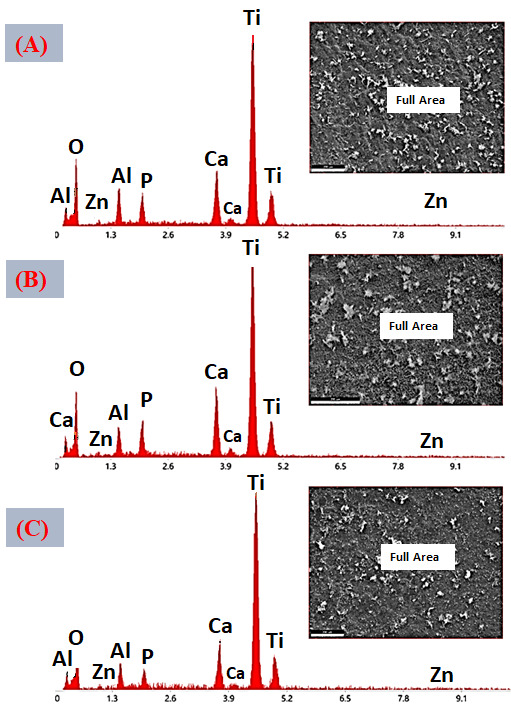
EDS spectra of the surfaces of Ti6Al4V coated with (**A**) VDZn1, (**B**) VDZn2, and (**C**) VDZn3.

Figure 16A–C presents the EDS spectra obtained from the surfaces coated with VDZn1, VDZn2, and VDZn3, respectively. In all cases, peaks corresponding to Ca, P, and O are clearly identified, confirming the formation of a calcium phosphate layer. Signals from Ti and Al originate from the Ti6Al4V substrate and indicate that the electron beam partially interacts with the underlying alloy, particularly in regions where the coating is thinner. Importantly, a Zn signal is detected in all variants, confirming successful incorporation of zinc into the coating through the biomimetic process.

The semi-quantitative EDS results (Table 4) reveal a progressive increase in atomic Zn content with increasing zinc concentration in the deposition solution: 0.4 at% for VDZn1, 0.7 at% for VDZn2, and 0.9 at% for VDZn3. This trend demonstrates that the method enables controlled zinc incorporation. Although the absolute Zn content remains relatively low, even trace levels of zinc are known to significantly influence osteogenic differentiation and antibacterial activity due to their role as an essential cofactor in numerous enzymatic processes [85].

**Table 4:** Semi-quantitative area EDS analysis of Zn-containing coating variants (atomic %).

Deposition Variant	Elements (at%)						
	C	O	Al	P	Ca	Ti	Zn
VDZn1	24.2	51.5	2.4	1.6	3.3	16.6	0.4
VDZn2	22.4	51.6	2.0	1.9	4.2	17.2	0.7
VDZn3	21.5	51.6	2.1	1.2	3.1	19.6	0.9

A slight increase in Ca and P content, particularly for VDZn2, suggests the existence of an optimal zinc concentration window in which apatite nucleation and Zn^2+^ incorporation occur simultaneously without severely inhibiting CaP growth. Moderate Zn levels may enhance surface reactivity by modifying crystal surface energy, potentially facilitating interaction with physiological fluids [86]. In contrast, excessive Zn appears to reduce overall CaP deposition efficiency, as reflected by the relative compositional changes and reduced surface coverage observed in VDZn3.

**Figure 17:**
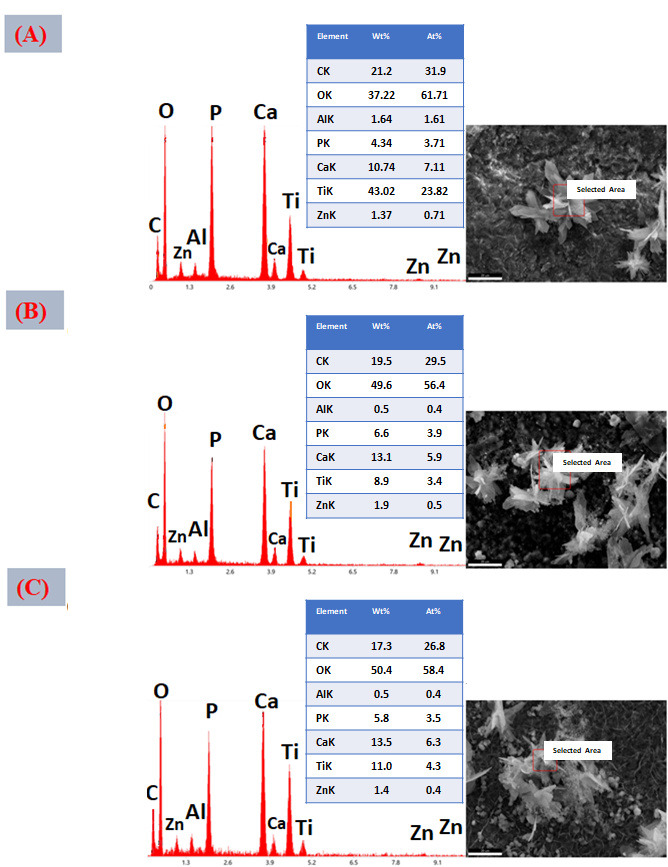
Point EDS analysis of the surfaces of Ti6Al4V coated with (**A**) VDZn1, (**B**) VDZn2, and (**C**) VDZn3.

Point EDS analysis (Figure 17A–C, VDZn1, VDZn2, and VDZn3, respectively) confirms that the flower-like structures are rich in Ca, P, and O, consistent with apatite precipitates. The simultaneous detection of Zn within these regions indicates that zinc is directly associated with the CaP phase. This association may occur through partial substitution of Ca^2+^ within the apatite lattice, interstitial incorporation, or surface adsorption onto crystal facets. Each mechanism can influence dissolution behavior and ion-release kinetics, which are critical parameters for achieving a balanced osteogenic and antibacterial response [87].

The Ca/P atomic ratio provides valuable insight into the chemical nature and structural maturity of the calcium phosphate phase formed in each Zn-containing coating. For reference, stoichiometric hydroxyapatite (HA) exhibits a Ca/P ratio of approximately 1.67. Deviations from this value often indicate the presence of non-stoichiometric phases, such as calcium-deficient apatite (CDA), amorphous calcium phosphate (ACP), or mixtures of different CaP phases [88]. These variations are particularly relevant in biomimetic systems, where precipitation typically occurs under near-physiological conditions and rarely yields perfectly stoichiometric HA. In the present study (Table 5), the Ca/P ratios are 1.44 for VDZn1, 1.51 for VDZn2, and 1.80 for VDZn3, revealing distinct compositional trends as a function of zinc concentration.

The Ca/P ratio of 1.44 (VDZn1) is clearly below the stoichiometric HA value. Such a reduced ratio suggests the formation of a calcium-deficient apatite or the presence of amorphous calcium phosphate as a precursor phase. In biomimetic deposition processes, ACP frequently forms during the early stages of nucleation and subsequently transforms into poorly crystalline apatite. A Ca/P ratio in the range of 1.3–1.5 is commonly associated with these metastable or calcium-deficient structures [89]. From a biological standpoint, calcium-deficient apatite is generally more soluble than stoichiometric HA. This higher solubility can enhance ion exchange with the surrounding environment, potentially promoting faster apatite reprecipitation in simulated body fluid and improved in vivo bioactivity. The relatively low zinc content (0.3 at%) in VDZn1 likely allows CaP nucleation to proceed with minimal structural disruption, resulting in a coating that retains the typical characteristics of biomimetic apatite, albeit in a slightly Ca-deficient form [90].

The Ca/P ratio increases to 1.51 in VDZn2, approaching more closely the stoichiometric HA value but still remaining within the calcium-deficient apatite range. This intermediate ratio is particularly interesting because it suggests a more balanced incorporation of calcium and phosphate during crystal growth. The moderate Zn content (0.5 at%) may contribute to this effect. Zinc ions are known to influence nucleation kinetics and crystal growth by interacting with active sites on growing CaP nuclei. At moderate concentrations, Zn^2+^ may slightly retard crystal growth without completely suppressing Ca incorporation, allowing a more controlled precipitation process [91]. The result is a composition closer to that of crystalline apatite while still retaining some degree of calcium deficiency. From a functional perspective, a Ca/P ratio around 1.5 is often considered advantageous in biomedical coatings. Calcium-deficient apatite with this composition tends to combine adequate structural stability with enhanced biological reactivity. It can dissolve gradually under physiological conditions, releasing Ca^2+^ and Zn^2+^ ions that may stimulate osteoblastic activity while maintaining sufficient coating integrity.

In contrast, VDZn3 exhibits a Ca/P ratio of 1.80, exceeding the stoichiometric HA value of 1.67. Such an elevated ratio suggests either a relative deficiency in phosphorus, an excess of calcium-rich phases, or the coexistence of secondary calcium-containing compounds (e.g., CaO or other Ca-rich precipitates) [92]. It may also reflect partial inhibition of phosphate incorporation during crystal growth due to higher Zn^2+^ concentrations. At elevated zinc levels, Zn^2+^ can compete more strongly with Ca^2+^ and may interfere with the normal arrangement of phosphate tetrahedra in the apatite lattice. This disruption can alter precipitation kinetics, potentially leading to non-stoichiometric compositions or heterogeneous phase formation. The morphological observations for VDZn3 (reduced surface coverage and greater heterogeneity) are consistent with such compositional imbalance. From a biological standpoint, a Ca/P ratio significantly higher than 1.67 may influence dissolution behavior and mechanical stability [93]. Calcium-rich phases may exhibit different solubility profiles and could lead to less predictable ion-release kinetics. Moreover, compositional heterogeneity may generate localized differences in dissolution rate, which could affect long-term coating performance.

**Figure 18:**
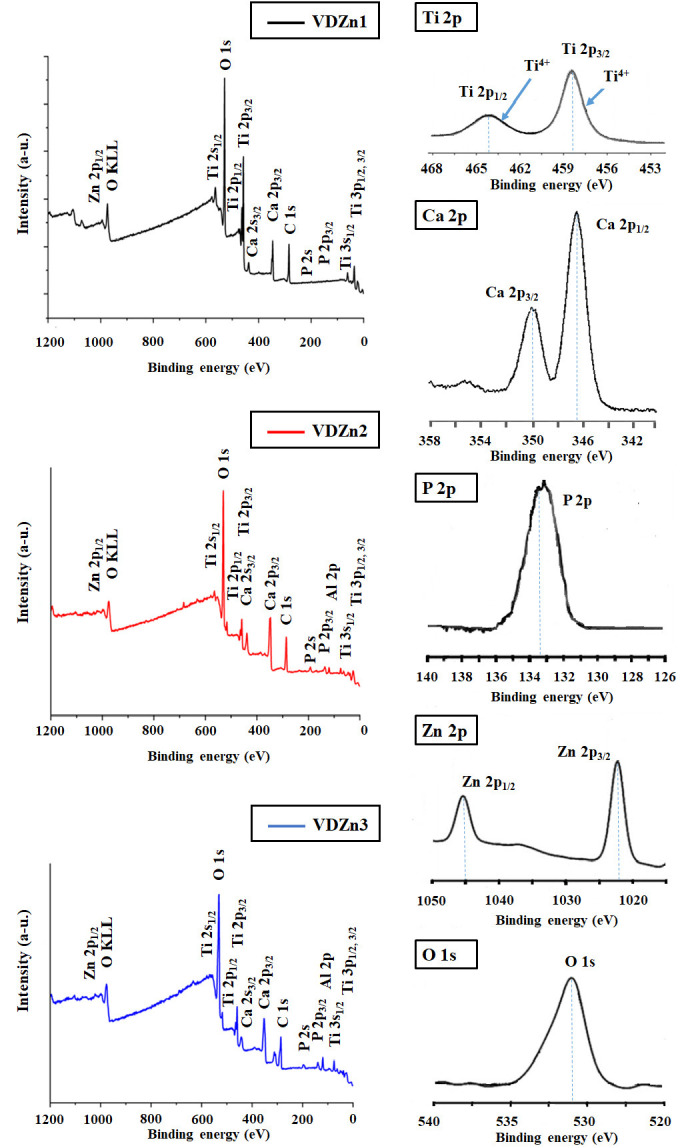
XPS energy analysis of Ti6Al4V surfaces coated with Zn-CaP.

To verify zinc incorporation and examine its chemical state at the outermost surface, X-ray photoelectron spectroscopy (XPS) was carried out (Figure 18).

The analysis confirmed the presence of Zn in all three variants, with surface atomic concentrations of 0.24%, 0.59%, and 0.93% for VDZn1, VDZn2, and VDZn3, respectively. These values are in very good agreement with the semi-quantitative EDS results, indicating consistent Zn incorporation both at the micrometric scale (EDS) and within the first few nanometers of the surface (XPS).

The high-resolution spectra (Ti 2p, O 1s, Ca 2p, P 2p, and Zn 2p) have been analyzed with clearer labeling of the corresponding chemical states. In particular, the Ti 2p region confirms the presence of Ti^4+^, consistent with a TiO_2_ surface layer. For the oxidation state Ti^4+^, the main peaks are found at Ti 2P_3/2_ (458.3 and 458.8 eV) and the Ti 2p_1/2_ peak (~463.9 eV–464.5 eV). Meanwhile, the Ca 2p and P 2p signals are characteristic of calcium phosphate phases. The two peaks in the Ca 2p region with binding energies of ~346.8 eV and ~350.3 eV are in perfect agreement with the characteristic positions of Ca 2p_1/2_ and Ca 2p_3/2_. The Zn 2p_3_/_2_ peak, centered at approximately ~1021–1022 eV, supports the presence of Zn^2+^ species, with no evidence of metallic zinc or other oxidation states. Additionally, the O 1s spectrum has been deconvoluted into contributions associated with lattice oxygen, hydroxyl groups, and adsorbed species, providing further insight into surface chemistry and potential nucleation sites.

The binding energies summarized in Table 6 are characteristic of calcium phosphate systems containing divalent zinc: (i) O1s (~529–531 eV), associated with O–P–O bonds and hydroxyl groups typical of hydroxyapatite; (ii) Ca2p (~346–347 eV), corresponding to Ca^2+^ in an apatite environment; (iii) P2p (~133.1 eV), characteristic of phosphate (PO_4_^3−^) groups; (iv) Zn2p (~1021–1022 eV), attributable to Zn^2+^ coordinated with oxygen in oxide or phosphate environments.

These values are consistent with previously reported data for zinc-doped hydroxyapatite and modified calcium phosphate coatings. The absence of significant shifts toward metallic Zn binding energies indicates that zinc is present in its oxidized state (Zn^2+^), rather than as metallic clusters [94]. Moreover, the relatively narrow variation in Ca2p and P2p positions among the three variants suggests that the overall apatite framework remains chemically stable despite the introduction of Zn. A closer look at the O1s signal reveals a slight shift toward lower binding energies as Zn content increases (from 531.1 eV in VDZn1 to 529.2 eV in VDZn3). This subtle change may reflect differences in the local chemical environment of oxygen, possibly due to Zn–O coordination or variations in hydroxyl content within the apatite lattice. Similarly, small variations in the Ca2p peak positions could be related to minor lattice distortions induced by Zn incorporation, given the smaller ionic radius of Zn^2+^ compared with Ca^2+^. Even limited substitution can generate measurable electronic perturbations detectable by surface-sensitive techniques such as XPS [95].

The incorporation of zinc into the CaP coatings through the biomimetic route represents one of the central contributions of this study. Both EDS and XPS analyses confirm the successful introduction of small but measurable amounts of Zn in all variants. Importantly, XPS demonstrates that zinc is present as Zn^2+^ in an oxygen-coordinated environment, consistent with its chemical association with the calcium phosphate phase rather than simple surface contamination. This finding confirms that the biomimetic process enables effective chemical integration of zinc within the coating [96].

**Table 6:** XPS peak positions (binding energy, eV) for Zn–CaP coatings deposited on Ti6Al4V.

Deposition Variant	C1s (eV)	O1s (eV)	Ca2p (eV)	P2p (eV)	Zn2p (eV)	Zn (at%, Surface)
VDZn1	284.6	531.1	347.1	133.1	1021.9	0.24
VDZn2	284.6	530.0	346.5	133.1	1020.7	0.59
VDZn3	284.6	529.2	346.1	133.1	1021.3	0.93

Beyond confirming its presence, the results show that zinc actively influences the precipitation chemistry of calcium phosphates. The progressive variation in the Ca/P atomic ratio from VDZn1 to VDZn3 demonstrates that Zn concentration modifies phase development and crystal growth dynamics. At low and intermediate Zn levels (VDZn1 and VDZn2), the Ca/P ratios correspond to calcium-deficient apatite, a phase known for its higher solubility and enhanced biological reactivity compared with stoichiometric hydroxyapatite. Under these conditions, the typical apatite morphology and satisfactory surface coverage are largely preserved, indicating that moderate Zn incorporation does not disrupt the nucleation–growth balance of the biomimetic system.

Among the studied conditions, VDZn2 emerges as the most balanced formulation. It combines moderate zinc incorporation with a Ca/P ratio closer to apatite stoichiometry, suggesting controlled crystal growth without compromising phase stability. This compositional and microstructural balance is particularly relevant for biomedical applications. Calcium-deficient apatite can promote ion exchange and surface reactivity, while maintaining sufficient structural integrity to ensure coating stability. In contrast, the highest Zn concentration (VDZn3) shifts the composition toward a Ca-rich regime (Ca/P = 1.80), indicating altered precipitation dynamics and possible phase heterogeneity. This deviation correlates with reduced coating homogeneity and surface coverage, suggesting that excessive Zn begins to destabilize the optimal biomimetic deposition window [97].

From a biological perspective, the controlled presence of Zn in the CaP layer is highly significant. Zinc is an essential trace element involved in key enzymatic pathways related to bone metabolism. It stimulates osteoblastic differentiation, enhances alkaline phosphatase activity, and supports extracellular matrix mineralization. At the same time, Zn^2+^ exhibits antibacterial activity against common implant-associated pathogens such as Staphylococcus aureus and Escherichia coli, partly through membrane destabilization and interference with microbial metabolism. Zinc has also been reported to modulate the local inflammatory response, which may contribute to improved early-stage healing. By integrating zinc into an osteoconductive CaP matrix, these coatings are designed as multifunctional systems capable of simultaneously promoting osseointegration and reducing infection risk [98].

Although XPS confirms the chemical state of zinc at the surface, it does not fully resolve whether Zn^2+^ is incorporated substitutionally within the apatite lattice or predominantly adsorbed onto crystal surfaces. Because XPS is limited to the near-surface region and primarily provides information on oxidation state and local chemical environment, complementary structural studies are necessary to clarify the incorporation mechanism. X-ray diffraction with Rietveld refinement could reveal subtle changes in lattice parameters; FTIR or Raman spectroscopy could detect vibrational shifts associated with phosphate or hydroxyl groups; and ion release studies in physiological media would help determine dissolution behavior and Zn^2+^ release kinetics [99].

In summary, this study demonstrates that zinc incorporation through a biomimetic approach is not merely a compositional modification but a strategy to tune microstructure, phase composition, and biological functionality. The results highlight the existence of an optimal Zn concentration range (represented here by VDZn2) where structural stability, controlled ion release, enhanced bioactivity, and antibacterial potential can be achieved simultaneously. This balance underscores the scientific and practical relevance of the present research for the development of next-generation multifunctional implant coatings.

#### 3.4.1. The Titanium Oxide Layer After Surface Treatment

As is well known, the titanium oxide layer formed after surface treatment plays a critical role as an interfacial layer, directly influencing the nucleation, growth, adhesion, and overall performance of the biomimetic CaP coatings. In this study, the surface activation of Ti6Al4V substrates via acid etching (HCl/H_2_SO_4_ at 80 °C for 20 min) was specifically designed to modify both the surface topography and the chemistry of the native oxide layer. Although the oxide layer thickness was not directly measured as an individual parameter, its presence and functional characteristics were indirectly assessed through a combination of surface-sensitive techniques and coating behavior.

From a physicochemical viewpoint, acid etching partially removes the native passive film and promotes the rapid reformation of a thin, hydrated titanium oxide layer (primarily TiO_2_) upon exposure to air and aqueous media. This regenerated oxide layer is typically nanometric in thickness and is known to be amorphous or poorly crystalline, enriched with hydroxyl (–OH) groups. These surface hydroxyl groups are essential, as they act as active sites for ionic interaction and might serve as nucleation centers for calcium phosphate during immersion in SBF.

The chemical nature of this interfacial layer was evaluated by X-ray photoelectron spectroscopy (XPS), which confirmed the presence of Ti^4+^ species consistent with TiO_2_, along with surface hydroxylation. Previous studies have demonstrated that such hydroxylated TiO_2_ surfaces significantly enhance apatite nucleation by increasing surface energy and facilitating electrostatic interactions with Ca^2+^ and PO_4_^3−^ ions.

Regarding morphology, the acid treatment generated a micro-roughened surface, as confirmed by SEM analysis. This topographical modification is intrinsically linked to oxide layer formation, as it increases the effective surface area and promotes mechanical interlocking with the subsequently deposited CaP coating.

While we acknowledge that direct measurement of oxide layer thickness (e.g., via TEM or ellipsometry) and crystallographic characterization (e.g., grazing-incidence XRD) would provide additional insight, the combined evidence from surface chemistry (XPS), morphology (SEM), and coating growth behavior collectively supports the formation of a thin, hydroxylated TiO_2_ interlayer with high bioactivity. Additionally, the uniform and dense nucleation of CaP observed in SEM images provides strong indirect evidence but strong evidence of a chemically active and homogeneous oxide layer.

#### 3.4.2. The Thickness of Biomimetic Zn-Containing CaP Coatings (Zn-CaP)

**Figure 19:**
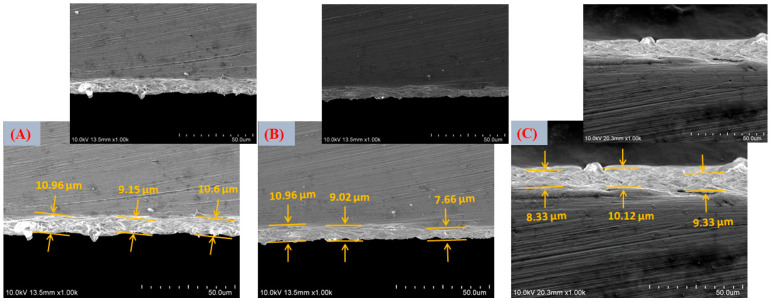
FESEM images of the thickness of Zn-CaP variants (and). (**A**) VDZn1, 1000×, (**B**) VDZn2, 1000×, and (**C**) VDZn3, 1000×.

Figure 19 shows the thickness of Zn-CaP variants (VDZn1, VDZn2, and VDZn3) determined through cross-sectional analysis using field emission scanning electron microscopy (FESEM). Representative cross-sectional micrographs were obtained, allowing direct visualization of the coating–substrate interface. The results indicate that the coatings exhibit a relatively uniform thickness, with average values in the range of approximately 9–11 µm. This thickness range is consistent with biomimetically deposited CaP layers obtained under similar conditions, where growth is governed by nucleation and ion diffusion processes in simulated body fluid (SBF). The observed uniformity also reflects the effectiveness of the prior surface activation treatment in promoting homogeneous nucleation across the substrate.

From a functional viewpoint, the measured thickness is sufficient to provide full surface coverage while maintaining good interfacial adhesion and avoiding excessive internal stresses. Moreover, coatings within this thickness range are known to support controlled ion release and bioactive behavior without compromising mechanical stability.

**Figure 20:**
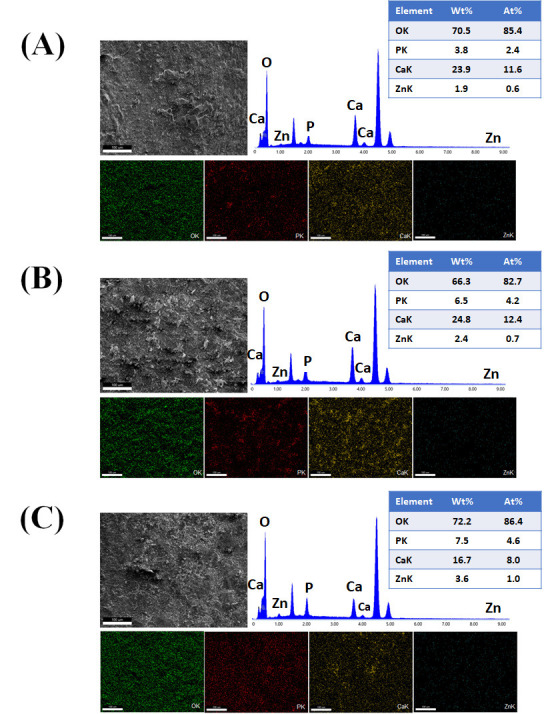
Elemental mapping study of Zn–CaP variants using FESEM–EDS. (**A**) VDZn1, (**B**) VDZn2, and (**C**) VDZn3.

To further validate the compositional homogeneity and Zn incorporation, the study was complemented by elemental mapping using FESEM–EDS for each Zn–CaP variant (see Figure 20). The EDS results (see Table 7) confirmed that all coatings are primarily composed of O, Ca, and P, consistent with calcium phosphate phases, with the additional presence of Zn in doped samples.

For VDZn1, the composition showed 70.3 wt% O, 23.9 wt% Ca, 3.8 wt% P, and 1.9 wt% Zn (0.6 at%), indicating successful incorporation of Zn at low levels. A similar compositional trend was observed for VDZn2, confirming the reproducibility of the biomimetic process and suggesting that Zn incorporation at intermediate conditions remains relatively stable without significantly altering the CaP matrix composition.

In contrast, VDZn3 exhibited a higher Zn content (3.6 wt%, 1.0 at%), accompanied by a decrease in Ca content (16.7 wt%) and an increase in oxygen concentration (72.2 wt%). This compositional shift suggests that higher Zn incorporation modifies the precipitation chemistry, potentially promoting the formation of more hydrated or defect-rich calcium phosphate phases. The reduction in Ca content, together with relatively stable P levels, indicates a deviation from stoichiometric apatite toward a more Ca-deficient or structurally perturbed phase, which is consistent with the Ca/P trends observed in previous sections. Elemental mapping further revealed a relatively homogeneous spatial distribution of Ca, P, and Zn across the coating thickness for all variants, with no evidence of Zn-rich agglomerates or phase segregation. This uniform distribution supports the hypothesis that Zn is incorporated both within the CaP matrix and at surface-accessible sites, rather than forming separate Zn-based phases.

From a microstructural perspective, the combination of uniform thickness and homogeneous elemental distribution indicates that the biomimetic deposition process enables controlled and reproducible incorporation of Zn without compromising coating continuity or integrity. However, the compositional variations observed at higher Zn levels (VDZn3) suggest that excessive Zn incorporation may alter crystal growth kinetics and local stoichiometry, which could influence both dissolution behavior and biological response. The integration of cross-sectional FESEM analysis with EDS compositional and mapping data provides a more comprehensive understanding of the structure–composition relationship in Zn–CaP coatings. These results confirm that coating thickness, elemental distribution, and Zn incorporation are strongly interrelated and must be carefully balanced to achieve optimal functional performance.

#### 3.4.3. The Adhesion of the Biomimetic Zn–CaP Coatings

**Figure 21:**
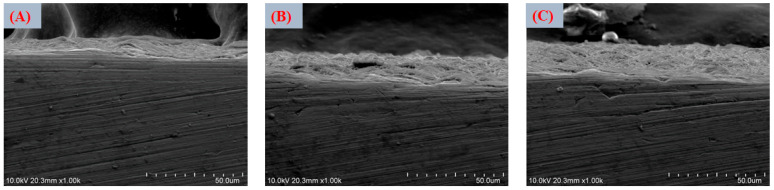
FESEM images of the adhesion stability of Zn-CaP variants after mechanical handling and ultrasonic cleaning. (**A**) VDZn1, 1000×, (**B**) VDZn2, 1000×, and (**C**) VDZn3, 1000×.

The adhesion of the biomimetic Zn–CaP coatings was qualitatively assessed through complementary observations of coating integrity after mechanical handling and ultrasonic cleaning. The coatings exhibited no signs of delamination, cracking, or spallation under these conditions, suggesting strong interfacial bonding with the Ti6Al4V substrate. Additionally, SEM cross-sectional analysis revealed a continuous and well-integrated interface without observable gaps or defects, which is typically associated with good adhesion in biomimetic systems (see Figure 21).

From a mechanistic perspective, the good adhesion can be attributed to two main factors: (i) the surface activation treatment, which generates a micro-roughened and hydroxylated TiO_2_ layer that enhances both mechanical interlocking and chemical bonding, and (ii) the low-temperature biomimetic deposition process, which promotes gradual nucleation and growth of CaP directly on the substrate, minimizing residual stresses commonly observed in other coating processes like plasma spraying. Further quantitative work needs to be addressed in order to study the mechanical properties of this coating.

Although a standardized quantitative adhesion test (e.g., pull-off test according to ASTM C633 (*Standard Test Method for Adhesion or Cohesion Strength of Thermal Spray Coatings*; ASTM International: West Conshohocken, PA, USA, 2013) or scratch test according to ASTM C1624-05 (*Standard Test Method for Adhesion Strength and Mechanical Failure Modes of Ceramic Coatings by Quantitative Single Point Scratch Testing*; ASTM International: West Conshohocken, PA, USA, 2005) was not initially included, the interface and coating morphologies obtained in this study are similar to those of other authors, who reported values of adhesion resistance in a range between ~10 and 30 MPa [100,101,102,103]. These values, while lower than those of some high-temperature coatings, are generally considered sufficient for dental implant applications, where load transfer is primarily supported by the metallic substrate and long-term stability is achieved through biological fixation (osseointegration) rather than solely by coating adhesion.

It is also important to note that excessively high adhesion strength is not always required or even desirable for bioactive coatings, as partial dissolution and remodeling at the interface are integral to their biological function. In this context, the combination of adequate initial adhesion and progressive bone bonding is more relevant for clinical performance.

### 3.5. Cytocompatibility

**Figure 22:**
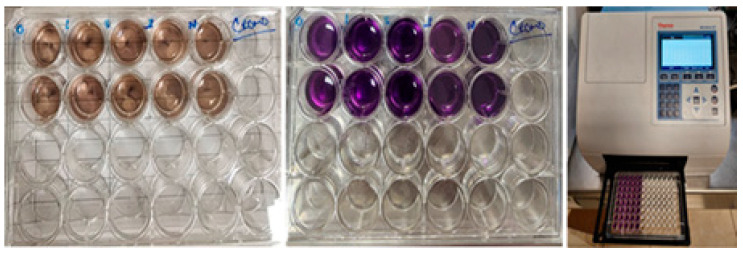
Change in the coloration of the cell culture that was in direct contact with the samples of the treatment variants VDZn0, VDZn1, VDZn2, and VDZN3. Microplate reader.

The cytotoxic behavior of the Zn–CaP coatings was evaluated using the MTT colorimetric assay, a widely accepted method that measures the ability of viable cells to reduce 3-(4,5-dimethylthiazol-2-yl)-2,5-diphenyltetrazolium bromide (MTT) into insoluble formazan crystals through NAD(P)H-dependent mitochondrial oxidoreductase activity. Since the formation of formazan is directly linked to mitochondrial integrity and cellular metabolic function, this assay provides sensitive information about early cytotoxic effects and overall cell viability [104]. After 24 h of direct contact between the samples and the cells, a clear purple coloration of the culture medium was observed for all variants, including VDZn1, VDZn2, and VDZn3 (Figure 22).

**Figure 23:**
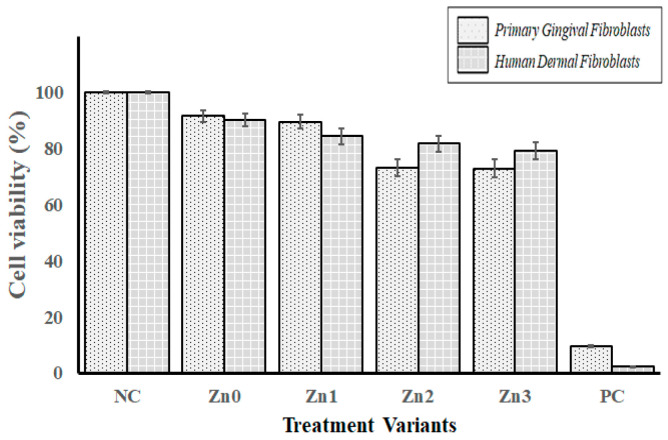
Cell viability percentage of Primary Gingival Fibroblasts (HGF-ATCC^®^ PCS-201-018TM) and Human Dermal Fibroblasts (ATCC^®^ PCS-2018-012) cultured in contact with the studied surfaces (NC-Negative Control, PC-Positive Control).

This qualitative change confirms efficient MTT reduction and indicates that mitochondrial activity was preserved in both primary human gingival fibroblasts (HGF) and human dermal fibroblasts (HDF). Even in the presence of zinc, none of the coatings caused complete inhibition of cellular metabolism. Quantitative evaluation was subsequently performed by spectrophotometric measurement of formazan absorbance using a microplate reader. Cell viability percentages were calculated relative to negative (NC) and positive (PC) controls following standardized protocols. The results for primary human gingival fibroblasts (HGF) and human dermal fibroblasts (HDF) are summarized in Figure 23 and Table 8.

The undoped CaP coating (VDZn0) exhibited high viability values (approximately 90–92%), confirming the excellent biocompatibility of calcium phosphate materials and aligning with the well-documented cytocompatibility of biomimetic apatite coatings [105]. When zinc was incorporated at low levels (VDZn1), cell viability remained high (>85%), indicating that small amounts of Zn^2+^ do not induce significant cytotoxic effects. This behavior is consistent with previous studies reporting that zinc, at controlled concentrations, can even exert beneficial biological effects.

**Table 8:** Cell viability (%) determined by MTT assay after 24 h of direct contact with Zn–CaP coatings.

Deposition Variant	Gingival Fibroblasts (HGF) (%) ± SD	Dermal Fibroblasts (HDF) (%) ± SD
NC	100.00 ± 0.50	100.00 ± 0.50
VDZn0 (CaP)	91.68 ± 2.10	90.26 ± 2.35
VDZn1	89.55 ± 2.45	84.36 ± 2.80
VDZn2	73.19 ± 3.10	81.74 ± 2.95
VDZn3	72.86 ± 3.25	79.07 ± 3.05
PC	9.690 ± 0.40	2.260 ± 0.10

As an essential trace element, Zn^2+^ functions as an enzymatic cofactor and plays a role in regulating gene expression associated with mineralization and tissue regeneration. At low doses, it may stimulate alkaline phosphatase activity and support extracellular matrix formation. In contrast, the intermediate and high zinc variants (VDZn2 and VDZn3) showed moderate reductions in viability. Compared with the positive control, gingival fibroblasts exhibited relative decreases of approximately 26–27%, while dermal fibroblasts showed reductions of about 18–21%. This pattern reflects a clear dose-dependent cellular response to Zn^2+^, as widely described in the literature [106]. While zinc is essential at physiological levels, elevated local concentrations may increase Zn^2+^ release into the culture medium, potentially disturbing intracellular ionic balance. Excess Zn^2+^ has been associated with oxidative stress, mitochondrial dysfunction, and interference with metabolic pathways when present above optimal thresholds [43].

All experiments were performed in triplicate (*n* = 3), and the results are presented as means ± standard deviation (SD). Statistical analysis was carried out using one-way analysis of variance (ANOVA) to evaluate the effect of Zn incorporation on cell viability. Prior to ANOVA, data normality and homogeneity of variances were verified using the Shapiro–Wilk and Levene tests, respectively. Differences were considered statistically significant at *p* < 0.05. The ANOVA results revealed statistically significant differences among the experimental groups (*p* < 0.001). Post hoc comparisons were performed using Tukey’s test. As expected, the positive control (PC) showed significantly lower cell viability compared to all other groups (*p* < 0.001), confirming the sensitivity of the assay, while the negative control (NC) exhibited the highest viability values. No statistically significant differences were observed between NC and the Zn-free CaP coating (VDZn0) (*p* > 0.05), indicating excellent cytocompatibility of the base coating. Similarly, the low-Zn variant (VDZn1) did not show significant differences compared to VDZn0 (*p* > 0.05), suggesting that low Zn incorporation does not adversely affect cell viability. In contrast, the intermediate and high Zn-containing coatings (VDZn2 and VDZn3) exhibited a statistically significant reduction in cell viability compared to NC and VDZn0 (*p* < 0.05), confirming a dose-dependent effect of Zn^2+^ on cellular response. However, no statistically significant difference was found between VDZn2 and VDZn3 (*p* > 0.05), indicating a plateau in the cytotoxic effect at higher Zn concentrations. Despite this reduction, all Zn-containing samples maintained viability values above the 70% threshold defined by ISO 10993-5 (*Biological Evaluation of Medical Devices—Part 5: Tests for In Vitro Cytotoxicity*; International Organization for Standardization (ISO): Geneva, Switzerland, 2009), confirming that the coatings remain non-cytotoxic within the evaluated range. The relatively low standard deviation values (<5%) further support the reproducibility and reliability of the MTT assay results.

Importantly, all Zn-containing coatings maintained viability values above 70% for both cell lines. According to ISO 10993-5 (*Biological Evaluation of Medical Devices—Part 5: Tests for In Vitro Cytotoxicity*; International Organization for Standardization (ISO): Geneva, Switzerland, 2009) criteria, materials with cell viability above this threshold are considered non-cytotoxic or slightly cytotoxic. Therefore, despite the observed dose-dependent reduction, the Zn–CaP coatings developed in this study can still be regarded as cytocompatible within the evaluated range.

Taken together, these findings highlight a critical balance in the design of multifunctional implant coatings. On one hand, zinc incorporation is intended to provide antibacterial properties and potentially enhance osteogenic responses; on the other hand, excessive Zn^2+^ release may compromise cellular viability. The results indicate that moderate zinc incorporation (particularly in VDZn1 and potentially VDZn2) offers a favorable compromise. These variants combine high or acceptable cell viability with effective Zn incorporation, preserving the biological compatibility of the CaP matrix while introducing the possibility of antibacterial and bioactive functionality.

This balance between biological safety and functional enhancement is central to the contribution of the present work. By demonstrating controlled Zn incorporation, dose-dependent cellular responses, and compliance with international cytotoxicity standards, the study establishes a solid foundation for subsequent in vitro bioactivity and antibacterial evaluations, where controlled Zn^2+^ release is expected to promote apatite formation while limiting bacterial colonization at the implant–tissue interface [107].

As is well known, cellular response in direct-contact cytotoxicity tests is governed by a combination of factors, including not only Zn^2+^ release but also surface-related properties such as roughness, surface chemistry/charge, wettability, and stiffness.

The acid-etched Ti6Al4V substrates exhibit increased micro-scale roughness and a hydroxylated TiO_2_ surface, which are known to enhance protein adsorption and initial cell attachment. After biomimetic deposition, the CaP coatings present a moderately rough, porous morphology and a hydrophilic character, both of which favor cell adhesion and spreading. At the same time, the incorporation of Zn^2+^ modifies surface chemistry and can influence local surface charge and ion exchange at the interface. Therefore, the observed cellular behavior is interpreted as the result of a coupled effect: favorable topographical and physicochemical features that promote adhesion, together with Zn^2+^ release that may modulate cell metabolism in a dose-dependent manner.

### 3.6. Antibacterial Activity

The antibacterial behavior of Ti6Al4V substrates coated with calcium phosphate (CaP) layers, with and without zinc incorporation, was evaluated using a standard plate counting method. The Gram-positive bacterium *Streptococcus mutans* was selected as the model microorganism due to its well-established role in oral biofilm formation and its clinical relevance in dental and implant-associated infections. This strain is characterized by strong surface adhesion, acidogenic metabolism, and a high capacity for early-stage colonization on biomaterials, making it particularly suitable for assessing the antibacterial performance of functionalized coatings [108].

**Figure 24:**
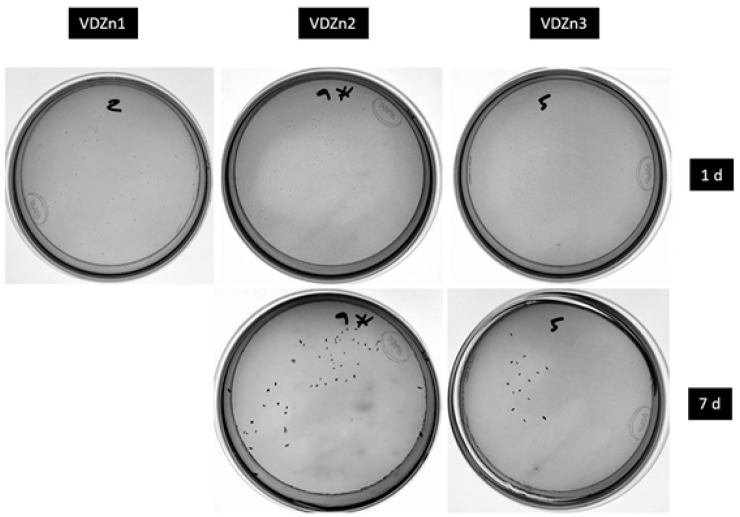
Bacterial growth of *S. mutans* colonies in contact with treatment variants VDZn1, VDZn2, and VDZn3 after one day of inoculation on agar. VDZn2* and VDZn3* observed after 7 days of incubation.

After 24 h of incubation on nutrient agar, the number of bacterial colonies formed on each Petri dish was carefully quantified. Both original plates and their replicas were analyzed to ensure reproducibility and statistical reliability. Representative images of bacterial growth at the highest dilution are shown in Figure 24.

In addition, images corresponding to prolonged incubation (7 days) for VDZn2* and VDZn3* are included to qualitatively evaluate the persistence of the antibacterial effect over time.

**Figure 25:**
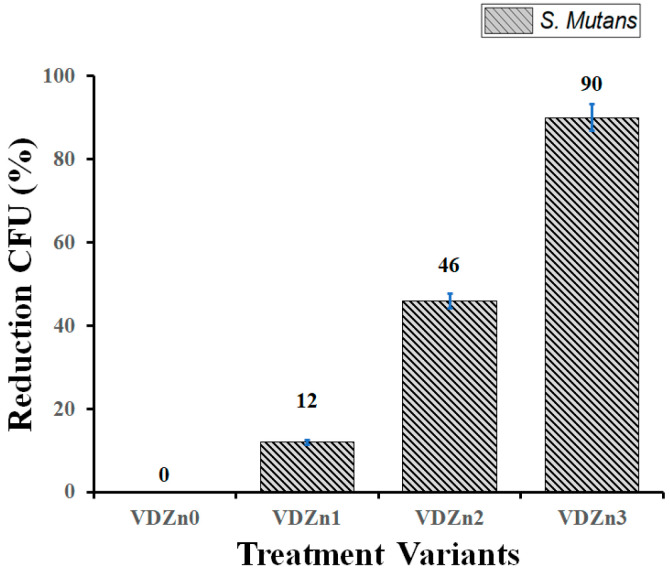
Percentage reduction of *S. mutans* colonies 24 h after being plated on nutrient aga. VDZn0 was used as the control surface.

The average colony number for each treatment (VDZn0, VDZn1, VDZn2, and VDZn3) was used to calculate colony-forming units (CFU) according to Equation (2), and the results are summarized in Table 9. A clear and progressive decrease in CFU values was observed as the zinc content in the coating increased, from 76 × 10^7^ CFU for the Zn-free CaP control (VDZn0) to 40 × 10^7^ CFU for the highest Zn-containing variant (VDZn3). When expressed as a percentage reduction relative to the control surface (Figure 25), the data reveal a well-defined concentration-dependent antibacterial response.

**Table 9:** Colony-forming units by treatment variant.

Deposition Variants	CFU ± SD
VDZn0	(76 ± 2.5) × 10^7^
VDZn1	(68 ± 2.4) × 10^7^
VDZn2	(52 ± 2.1) × 10^7^
VDZn3	(40 ± 1.8) × 10^7^

All antibacterial experiments were performed in triplicate (*n* = 3) using independent samples, and the results are expressed as means ± standard deviation (SD). Colony-forming units (CFU) were quantified after incubation, and statistical analysis was carried out using one-way analysis of variance (ANOVA) to evaluate the effect of Zn incorporation on bacterial viability. Prior to ANOVA, the assumptions of normality and homogeneity of variances were verified using the Shapiro–Wilk and Levene tests, respectively. Differences were considered statistically significant at *p* < 0.05. The ANOVA results indicated statistically significant differences in CFU values among the different coating variants (*p* < 0.05). Post hoc multiple comparisons were performed using Tukey’s test to identify specific differences between groups. The Zn-free CaP coating (VDZn0) exhibited significantly higher CFU values compared to all Zn-containing variants (*p* < 0.01), confirming the antibacterial effect of Zn incorporation. Furthermore, a progressive and statistically significant reduction in CFU was observed with increasing Zn content from VDZn1 to VDZn3 (*p* < 0.05), demonstrating a clear dose-dependent antibacterial response. Significant differences were also identified between intermediate and high Zn concentrations (VDZn2 vs. VDZn3, *p* < 0.05), indicating that higher Zn levels further enhance bacterial inhibition. The relatively low standard deviation values (<5%) confirm good experimental reproducibility and reliability of the microbiological assay, supporting the robustness of the observed antibacterial trends.

Zn-containing coatings therefore exhibited a clear, dose-dependent antibacterial effect against *S. mutans*. The systematic reduction in viable colonies confirms that zinc incorporation effectively imparts antibacterial functionality to the CaP matrix. This behavior is widely attributed to the controlled release of Zn^2+^ ions, which can disrupt bacterial membrane integrity, interact with thiol groups in essential metabolic enzymes, induce intracellular oxidative stress, and inhibit biofilm formation and maturation [109].

In the specific case of *S. mutans*, Zn^2+^ has been reported to interfere with glycolytic pathways and acid production, both of which are critical for its cariogenic activity and adhesion capacity. Zinc can also impair extracellular polysaccharide synthesis, reducing biofilm stability and limiting bacterial colonization on implant surfaces. These mechanisms are particularly relevant in dental and orthopedic applications, where early bacterial adhesion is a decisive factor in peri-implant infection.

At the same time, an inverse relationship between antibacterial activity and cell viability was observed as the zinc content increased. While higher Zn incorporation enhanced bacterial inhibition, it also led to a moderate decrease in fibroblast viability, although all values remained above the 70% threshold commonly accepted for non-cytotoxic materials. This trade-off between antimicrobial efficacy and cytocompatibility is consistent with previous studies on metal-ion-functionalized CaP systems, where an optimal zinc concentration range provides antibacterial benefits while preserving acceptable host cell responses [110].

From a mechanistic perspective, Zn^2+^ release kinetics play a central role in balancing these biological effects. Not only the total zinc content, but also its distribution within the apatite lattice, chemical state, and degree of substitution for Ca^2+^ influence the effective concentration of Zn^2+^ at the interface. Structural incorporation within the apatite network tends to favor a more sustained and controlled release profile, whereas loosely bound zinc may lead to a faster initial release, enhancing antibacterial performance but increasing the risk of cytotoxic effects. Therefore, precise control over zinc incorporation is essential for optimizing multifunctional coatings [111].

Among the evaluated variants, VDZn3 exhibited the strongest antibacterial effect, whereas VDZn1 provided the highest level of cytocompatibility with moderate antibacterial activity. In this context, VDZn2 appears to represent the most balanced configuration, combining a substantial reduction in CFU with acceptable cell viability.

These findings support the design strategy of multifunctional CaP-based implant coatings in which controlled zinc incorporation enables simultaneous osteoconductive and anti-infective properties. The results also provide a solid framework for correlating ion release behavior, biomimetic apatite formation, and antibacterial performance, contributing to the rational development of implant surfaces that promote tissue integration while minimizing infection risk.

### 3.7. Zn Ion Release into the Surrounding Medium

#### 3.7.1. Physicochemical Basis of Ion Release in CaP Coatings

Calcium phosphate (CaP) coatings obtained by biomimetic routes are typically characterized by a partially crystalline and often carbonated apatite structure, closely resembling the mineral phase of bone. This structural similarity is not only compositional but also functional, as it confers high surface energy and a strong tendency to interact with ionic species present in physiological environments [112]. In aqueous media, these coatings do not behave as inert layers; instead, they participate in a dynamic dissolution–reprecipitation process. The initial release of calcium and phosphate ions increases local supersaturation, which in turn promotes the nucleation of a secondary apatite layer with improved structural organization. This sequence is widely regarded as a crucial indicator of in vitro bioactivity and is often correlated with the ability of a material to bond to living bone.

When Zn^2+^ is introduced into the CaP system, its incorporation is not restricted to a single structural position. Depending on synthesis conditions and local chemistry, zinc may substitute for Ca^2+^ within the apatite lattice, be trapped within amorphous calcium phosphate domains, or remain weakly bound at the surface through electrostatic interactions with phosphate and hydroxyl groups. The smaller ionic radius of Zn^2+^ compared with Ca^2+^ generates local lattice distortions, which tend to reduce crystallinity and increase defect density. From a thermodynamic standpoint, this destabilization raises the free energy of the solid phase, making it more prone to dissolution under physiological conditions [113].

This effect is particularly relevant in biomimetic coatings, which already exhibit a relatively low degree of crystallinity. In such systems, even small compositional changes can significantly alter dissolution kinetics. As a result, Zn-containing CaP coatings often display enhanced ion release compared with their undoped counterparts. Importantly, this increased solubility is not necessarily detrimental; rather, it can be advantageous when it enables a controlled and sustained release of biologically active ions [114]. In the present case, Zn^2+^ release becomes a decisive parameter linking the structural features of the coating with its biological performance.

#### 3.7.2. Zinc Release Kinetics and Coupling with Biomimetic Bioactivity

The release of Zn^2+^ into a physiological medium (PBS) revealed a consistent trend across all compositions, characterized by an early-stage release followed by a progressive reduction in ion flux. This type of profile is commonly reported for ion-doped calcium phosphates and reflects the heterogeneous distribution of dopant species within the coating [115].

**Figure 26:**
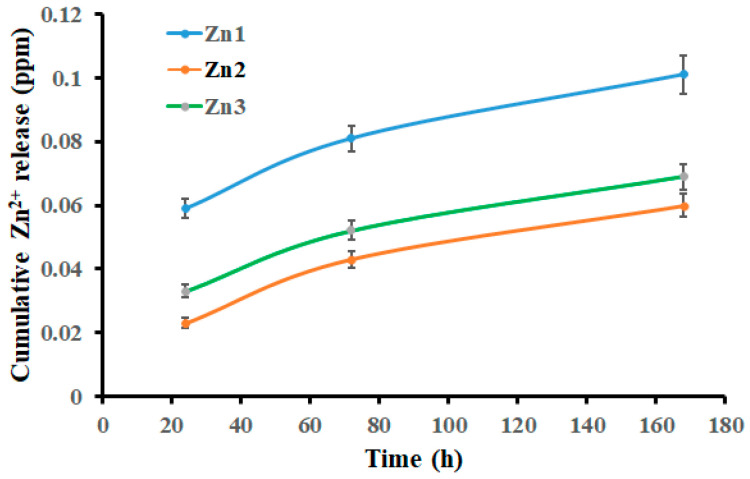
Zn^2+^ release profile in 50 mL phosphate-buffered saline (PBS).

The Zn^2+^ release profiles for the VDZn1, VDZn2, and VDZn3 are shown in Figure 26. A notable observation is that the sample with the lowest nominal zinc content (VDZn1) exhibited the highest Zn^2+^ concentration at 24 h.

This apparent inconsistency suggests that zinc is not uniformly incorporated into the apatite structure. Instead, a significant fraction of Zn in VDZn1 is likely present in surface-accessible or weakly bound states, which are rapidly released upon immersion [116]. In contrast, samples with higher zinc content (VDZn2 and VDZn3) may contain a larger proportion of structurally incorporated Zn, resulting in a slower and more diffusion-controlled release.

After 72 h, the convergence of Zn^2+^ concentrations across all samples indicates that the contribution of easily exchangeable species becomes negligible, and the release mechanism transitions toward one governed by matrix dissolution. The stabilization observed after 7 days further supports this interpretation, suggesting the establishment of a quasi-equilibrium between dissolution and reprecipitation processes at the coating–solution interface. Table 10 shows the cumulative Zn^2+^ release (mg·L^−1^) in PBS at different incubation times.

Statistical analysis was performed using one-way analysis of variance (ANOVA) to evaluate the effect of Zn concentration and incubation time on Zn^2+^ release. Prior to ANOVA, data normality and homogeneity of variances were verified using the Shapiro–Wilk and Levene tests, respectively. All experiments were conducted in triplicate (*n* = 3), and results are presented as means ± standard deviations. The ANOVA results revealed statistically significant differences (*p* < 0.05) in Zn^2+^ release among the different coating variants (VDZn1, VDZn2, and VDZn3) at each incubation time. Post hoc multiple comparisons were performed using Tukey’s test to identify specific differences between groups. At 24 h, VDZn1 exhibited significantly higher Zn^2+^ release compared to VDZn2 (*p* < 0.01) and VDZn3 (*p* < 0.05), confirming the presence of a higher fraction of surface-accessible Zn species. At 72 h and 168 h, the differences between VDZn2 and VDZn3 were reduced and, in some cases, not statistically significant (*p* > 0.05), indicating convergence toward a diffusion-controlled release regime. Additionally, a two-way ANOVA considering both Zn concentration and incubation time as independent factors confirmed that both variables, as well as their interaction, had a statistically significant effect on Zn^2+^ release (*p* < 0.05). This result supports the interpretation that ion release is governed by a coupled mechanism involving both compositional and kinetic factors. These statistical findings reinforce the reliability of the observed trends and confirm that the differences in Zn^2+^ release are not due to random variability but are intrinsically linked to the physicochemical characteristics of the coatings.

The cumulative release values reinforce this interpretation, showing that total Zn^2+^ release is not directly proportional to the initial zinc content. This behavior highlights the importance of considering not only composition but also the physicochemical state of the dopant. From a design perspective, it suggests that optimizing Zn functionality requires precise control over its spatial distribution within the coating rather than simply increasing its concentration [117].

The release behavior can be described by a two-stage mechanism: (i) a rapid initial release dominated by desorption and dissolution of loosely bound Zn species, and (ii) a slower stage controlled by diffusion through the CaP matrix and its gradual degradation. This dual mechanism is particularly advantageous for biomedical applications, as it provides an early antibacterial effect followed by sustained ion delivery [118].

In this context, recent advances in surface engineering further support the relevance of coupling controlled degradation with functional ion release as a crucial design principle for next-generation implant coatings. Studies on plasma electrolytic oxidation (PEO)-based and composite coatings, particularly in magnesium systems, have demonstrated that the integration of gradient architectures and tailored microstructures enables a fine regulation of degradation kinetics and therapeutic ion delivery. These systems highlight that the biological performance of a coating is not solely dictated by its composition but also by the dynamic interplay between dissolution behavior, ion release rates, and interfacial reactions with the surrounding environment. Although such approaches have been primarily explored in biodegradable substrates, the same principles are directly applicable to biomimetic CaP coatings. In the present study, the observed two-stage Zn^2+^ release mechanism and the establishment of a quasi-equilibrium state are consistent with these concepts, where controlled degradation governs both antibacterial activity and bioactive response. Therefore, the results reinforce the idea that optimizing coating performance requires precise control over both structural features and ion release kinetics, rather than simply increasing dopant concentration [35,36].

#### 3.7.3. Biological Implications of Zn^2+^ Release

The biological response associated with Zn^2+^ release reflects a balance between its beneficial and potentially adverse effects. On one hand, zinc is well known for its antimicrobial activity. Its action is not limited to a single pathway; rather, it involves membrane destabilization, interference with enzyme activity through binding to thiol groups, and the generation of oxidative stress within bacterial cells. These combined effects impair bacterial viability and reduce their ability to form stable biofilms.

In the case of *Streptococcus mutans*, a crucial microorganism in oral biofilm formation, Zn^2+^ can disrupt carbohydrate metabolism and reduce acid production, which in turn affects adhesion and colonization. This mechanism is particularly relevant for implant-related infections, where early bacterial attachment plays a critical role in the establishment of biofilms [119].

The experimental results obtained here are consistent with these mechanisms. The reduction in colony-forming units, especially for the VDZn3 condition, indicates that the local concentration of Zn^2+^ reached levels sufficient to inhibit bacterial growth. At the same time, the absence of excessively high release suggests that cytotoxic thresholds were not exceeded.

Nevertheless, the observed decrease in cell viability with increasing zinc content points to a narrow therapeutic window [120]. While moderate Zn^2+^ levels can stimulate osteogenic activity and improve antibacterial performance, excessive concentrations may impair cell proliferation. This highlights the need for careful optimization of Zn incorporation, ensuring that release rates remain within a range that supports tissue integration while preventing bacterial colonization.

These observations are consistent with recent advances in multifunctional coating design, where the synergy between material degradation and biological response has been identified as a critical factor governing implant performance. In particular, studies on plasma electrolytic oxidation (PEO)-based coatings in magnesium systems have demonstrated that corrosion-driven ion release can be strategically tuned to simultaneously promote antibacterial activity and tissue regeneration. Although the present system is not based on biodegradable metals, a clear parallel can be established: in Zn–CaP coatings, dissolution-mediated Zn^2+^ release plays an analogous role to corrosion processes in magnesium alloys. In both cases, the biological outcome is dictated by the kinetics of ion release and the resulting local microenvironment at the interface. This comparison highlights that achieving an optimal balance between antimicrobial efficacy and cytocompatibility requires precise control over degradation behavior and ion delivery rates. Therefore, the results of this study further support the concept that the design of bioactive coatings should be approached from a dynamic perspective, where controlled material transformation and ion release are intentionally engineered to modulate biological responses [35,36].

### 3.8. Surface Roughness Evolution and Its Implications

Surface roughness plays a critical role in determining the biological performance of titanium-based implants, as it directly influences protein adsorption, cell attachment, and subsequent tissue integration. In the present study, roughness measurements were carried out using a contact profilometer to quantify the topographical changes induced by sequential surface treatments and subsequent Zn–CaP coating deposition.

#### 3.8.1. Surface Roughness After Pre-Treatments

The evolution of surface roughness closely reflects the morphological and compositional transformations induced by each modification step, revealing a progressive transition from a relatively smooth and anisotropic surface toward a more complex and functionally active interface. Table 11 summarizes the evolution of surface roughness parameters (Ra, Rz, and Rq) following each surface pre-treatment step.

The as-machined Ti6Al4V substrate exhibited the lowest roughness values (Ra ≈ 0.163 µm), consistent with its characteristic anisotropic topography defined by machining grooves. Such a surface provides limited effective area and a low density of active sites, which restricts its interaction with the physiological environment and reduces its ability to promote biological anchorage.

Following acid etching, a marked increase in roughness was observed (Ra ≈ 0.490 µm), associated with the formation of a homogeneous network of micropits and valleys. This treatment effectively removes machining marks while introducing a microstructured surface with enhanced peak-to-valley amplitude. From a physicochemical standpoint, this modification is particularly relevant, as it increases the available surface area and promotes heterogeneous nucleation by creating energetically favorable sites for subsequent reactions.

**Table 11:** Surface roughness parameters (Ra, Rz, and Rq) of Ti6Al4V substrates after sequential surface pre-treatments. Values are reported as means ± standard deviations (*n* = 3). Ra: arithmetic mean roughness; Rz: average maximum height of the profile; Rq: root mean square roughness.

Treatment	Ra (µm) ± SD	Rz (µm) ± SD	Rq (µm) ± SD
As-machined	0.163 ± 0.034	1.313 ± 0.273	0.306 ± 0.275
Acid-etched	0.490 ± 0.023	3.812 ± 0.283	0.635 ± 0.026
NaOH-treated	0.365 ± 0.047	2.566 ± 0.509	0.471 ± 0.073
CaCl_2_-treated	0.477 ± 0.100	3.564 ± 0.741	0.615 ± 0.110
Heat-treated (TT)	0.551 ± 0.095	3.966 ± 0.761	0.718 ± 0.128

In contrast, the alkaline treatment in NaOH resulted in a moderate decrease in micrometric roughness (Ra ≈ 0.365 µm), despite generating a highly porous nanostructure. This apparent discrepancy suggests that the formation of a sodium titanate gel layer leads to partial smoothing at the micrometric scale while simultaneously increasing surface complexity at the nanoscale. This highlights an important limitation of conventional roughness parameters (Ra, Rz), which may not fully capture multiscale structural features. Rather than simply increasing roughness, the alkaline treatment primarily induces chemical activation, enriching the surface with reactive groups that play a key role in subsequent coating formation.

The incorporation of calcium through CaCl_2_ treatment led to a renewed increase in roughness (Ra ≈ 0.477 µm), accompanied by higher variability in the measured values. This behavior is consistent with the formation of localized calcium-rich precipitates, introducing surface heterogeneity and creating preferential nucleation sites for calcium phosphate deposition. These features indicate a transition toward a chemically and topographically activated surface.

The highest roughness values were obtained after thermal treatment (Ra = 0.551 ± 0.095 µm; Rz = 3.966 ± 0.761 µm), which can be attributed to the consolidation and stabilization of the previously formed gel-like layer. Thermal dehydration and structural contraction likely induce microcrack formation, further increasing surface complexity. These features not only enhance mechanical interlocking but also improve interfacial reactivity by exposing additional active sites.

Although the hydrothermal treatment (TH_2_O) was not evaluated in terms of roughness, its primary contribution is expected to be chemical rather than topographical. This step promotes ion exchange and the formation of surface hydroxyl groups (Ti–OH), which are known to enhance bioactivity and facilitate apatite nucleation without significantly altering micrometric roughness.

The roughness evolution does not follow a monotonic trend but rather reflects the interplay between dissolution, reorganization, and surface growth processes. More importantly, these results demonstrate that surface activation is not governed solely by roughness magnitude, but by the combined effect of topography and surface chemistry. The final treated surface, characterized by moderate-to-high roughness, chemical functionality, and hierarchical porosity, provides a suitable platform for biomimetic coating deposition.

A statistical analysis was performed to evaluate the significance of the differences observed in surface roughness parameters (Ra, Rz, and Rq) among the different surface treatments. One-way analysis of variance (ANOVA) was applied independently to each roughness parameter (Ra, Rz, and Rq), considering the treatment condition as the main factor (*n* = 3 per group). The results revealed that surface treatment had a statistically significant effect on all roughness parameters (*p* < 0.01 for Ra, Rz, and Rq), indicating that the modifications induced by each sequential treatment step are not due to random variation but to systematic changes in surface morphology.

Post hoc comparisons using the Tukey HSD test further showed that the acid-etched and heat-treated (TT) conditions exhibited significantly higher roughness values compared to the as-machined surface (*p* < 0.05) for all parameters. In particular, Ra increased from 0.163 ± 0.034 µm (as-machined) to 0.490 ± 0.023 µm (acid-etched) and 0.551 ± 0.095 µm (TT), confirming a substantial modification of the surface profile. Similarly, Rz and Rq followed the same trend, with statistically significant increases after chemical and thermal treatments.

No statistically significant differences (*p* > 0.05) were observed between some intermediate treatments (NaOH-treated and CaCl_2_-treated), suggesting that these steps contribute to surface modification in a more gradual manner. However, the cumulative effect of the sequential treatment, particularly after the final heat treatment, resulted in the highest roughness values, which are statistically distinguishable from the initial condition. These statistical results confirm that the applied surface treatments produce significant and reproducible modifications in surface roughness, supporting their role in enhancing surface reactivity and providing favorable conditions for subsequent biomimetic CaP deposition.

#### 3.8.2. Surface Roughness of Zn–CaP Coatings

Table 12 presents the surface roughness parameters of the Zn–CaP coatings as a function of Zn incorporation. The roughness of the Zn–CaP coatings reveals a clear relationship between surface topography, coating morphology, and chemical composition, highlighting the role of Zn as a regulator of biomimetic growth.

The Zn-free coating (VDZn0) exhibited the highest roughness values (Ra ≈ 0.75 µm), which correlates well with the formation of large, flower-like apatite structures observed by FESEM. This morphology is typically associated with high supersaturation conditions, where rapid and uncontrolled crystal growth leads to heterogeneous aggregation and increased surface irregularity.

The introduction of Zn at low concentration (VDZn1) resulted in a significant reduction in roughness (Ra ≈ 0.50 µm), accompanied by a refinement of the microstructure. Although the flower-like morphology was preserved, the particle size decreased and the distribution became more uniform. The presence of Zn, confirmed by EDS and XPS, suggests that Zn^2+^ ions begin to modify the surface energy of growing nuclei, acting as a kinetic regulator that limits excessive crystal growth without suppressing CaP formation.

This effect becomes more pronounced at intermediate Zn concentration (VDZn2), where the lowest roughness values were recorded (Ra = 0.39 ± 0.04 µm; Rz = 2.81 ± 0.24 µm). The corresponding microstructure is characterized by smaller, more fragmented aggregates and improved surface uniformity. Compositional analysis indicates higher Ca and P contents along with increased Zn incorporation, suggesting a condition in which nucleation and growth are balanced. The resulting coating is more compact and homogeneous, reflecting a more controlled crystallization process.

At higher Zn concentration (VDZn3), roughness increases again (Ra ≈ 0.72 µm), approaching the values observed for the Zn-free system. This behavior is consistent with a reduction in coating coverage and increased heterogeneity, as observed in SEM images. Although Zn content is higher, excessive Zn^2+^ appears to inhibit both nucleation and ordered growth of the CaP phase, leading to less compact and more irregular structures.

Taken together, these results demonstrate a non-linear relationship between Zn concentration and surface roughness, reflecting a transition in the coating formation mechanism. In the absence of Zn, growth is dominated by supersaturation-driven crystallization, resulting in coarse and heterogeneous structures. At low and intermediate concentrations, Zn acts as a growth modulator, promoting finer and more uniform coatings. At higher concentrations, however, Zn exerts an inhibitory effect, disrupting the crystallization process and leading to increased roughness.

From a functional perspective, this behavior is highly relevant. Surface roughness influences not only mechanical interlocking but also protein adsorption, cell attachment, and bacterial adhesion. In this context, the VDZn2 condition appears to provide an optimal balance between structural homogeneity, controlled roughness, and Zn incorporation. This combination is expected to favor bioactivity while maintaining antibacterial functionality, making it particularly suitable for implant surface applications.

A statistical analysis was conducted to assess the effect of Zn incorporation on the surface roughness parameters (Ra, Rz, and Rq) of the Zn–CaP coatings. One-way analysis of variance (ANOVA) was performed independently for each parameter, considering the coating variant (VDZn0, VDZn1, VDZn2, and VDZn3) as the main factor (*n* = 3 per group). The results demonstrated that Zn content has a statistically significant influence on all roughness parameters (*p* < 0.01 for Ra, Rz, and Rq), confirming that the observed variations are not due to experimental scatter but to systematic changes induced by Zn incorporation. Post hoc analysis using the Tukey HSD test revealed that the VDZn2 condition exhibited significantly lower roughness values compared to VDZn0 and VDZn3 (*p* < 0.05) for all parameters, indicating a more uniform and refined surface morphology at intermediate Zn levels. In particular, Ra decreased from 0.75 ± 0.07 µm (VDZn0) to 0.39 ± 0.04 µm (VDZn2), while Rz and Rq followed the same trend, supporting the interpretation of controlled crystal growth and improved coating homogeneity. In contrast, VDZn3 showed a statistically significant increase in roughness compared to VDZn2 (*p* < 0.05), with values approaching those of the Zn-free coating (VDZn0). This result suggests that excessive Zn incorporation disrupts the regular growth of the CaP phase, leading to increased surface heterogeneity and morphological irregularities. No statistically significant differences (*p* > 0.05) were observed between VDZn0 and VDZn3 for some parameters, indicating that high Zn content may partially revert the surface characteristics toward those of the undoped system. The statistical analysis confirms a non-linear relationship between Zn concentration and surface roughness, with VDZn2 representing the optimal condition in terms of minimizing roughness and achieving a more homogeneous surface. These findings reinforce the role of Zn as a key regulator of biomimetic growth and highlight the importance of controlling its concentration to tailor surface topography and functional performance.

#### 3.8.3. Structure–Property Relationship Connected with Surface Roughness

The surface roughness results exhibit a clear non-linear dependence on Zn incorporation, which can be consistently correlated with the microstructural evolution, chemical composition, ion release behavior, and biological performance of the coatings. In the absence of Zn (VDZn0), the high roughness values (Ra = 0.75 ± 0.07 µm) are associated with the formation of large, flower-like apatite aggregates, as observed by FESEM, resulting from an uncontrolled growth regime under high supersaturation. EDS and XPS analyses confirm a typical CaP composition with relatively higher crystallinity and lower defect density, which limits ion release and, consequently, provides no intrinsic antibacterial functionality despite potentially favorable conditions for initial protein adsorption.

The incorporation of Zn at low concentration (VDZn1) leads to a significant reduction in roughness (Ra = 0.50 ± 0.18 µm), accompanied by a refinement of the microstructure, with smaller and more uniformly distributed particles. XPS results indicate that Zn^2+^ exists both as structurally incorporated species and as surface-accessible ions, which explains the relatively high initial release observed at early immersion times. This dual chemical state promotes an initial antibacterial effect while preserving a surface topography that supports cell attachment and spreading.

At intermediate Zn concentration (VDZn2), the lowest roughness values are obtained (Ra = 0.39 ± 0.04 µm), reflecting the formation of a compact and homogeneous coating with improved surface coverage and reduced particle size, as confirmed by FESEM. EDS and XPS analyses further indicate a more effective incorporation of Zn within the CaP lattice, resulting in a more controlled and sustained Zn^2+^ release profile. This condition establishes an optimal balance between surface topography, chemical stability, and ion release kinetics, which is directly reflected in the biological response. Specifically, the combination of moderate roughness and homogeneous morphology promotes stable cell–surface interactions and cytocompatibility, while the controlled release of Zn^2+^ provides sufficient antibacterial activity without exceeding cytotoxic thresholds.

In contrast, further increasing the Zn content (VDZn3) leads to a renewed increase in roughness (Ra = 0.72 ± 0.08 µm), indicating a transition toward a less controlled growth mechanism. FESEM observations reveal reduced coating density and increased heterogeneity, while XPS confirms a higher fraction of surface-accessible Zn. This condition enhances antibacterial performance due to increased Zn^2+^ availability but also results in localized ion accumulation and surface irregularities that may negatively affect cell viability, highlighting the onset of a cytotoxic threshold.

These findings demonstrate that Zn acts not merely as a dopant but as a key regulator of nucleation kinetics, crystal growth, and defect formation, thereby governing surface roughness, Zn distribution, and ion release behavior. Importantly, the biological response arises from the synergistic interplay between topography, chemical composition, and release kinetics rather than from any single parameter. Within this framework, the VDZn2 condition represents an optimal “therapeutic window”, where microstructural uniformity, controlled Zn incorporation, and balanced antibacterial and cytocompatible responses are simultaneously achieved. This highlights the importance of integrated surface design strategies for the development of multifunctional implant coatings.

### 3.9. Current Applicability and Future Development Needs of the Proposed Coatings

The Zn-doped calcium phosphate (Zn–CaP) coatings developed in this study exhibit promising characteristics for biomedical applications, including homogeneous coverage, controlled Zn^2+^ release, antibacterial activity, and acceptable cytocompatibility. From a materials science perspective, these features indicate that the coatings are suitable candidates for enhancing the biological performance of Ti6Al4V implants, particularly in promoting early-stage osseointegration while reducing the risk of bacterial colonization. However, at the current stage, the coatings cannot yet be considered ready for direct clinical use on medical implants. While the in vitro results are encouraging, several critical aspects must be further investigated and optimized before clinical translation: (i) mechanical reliability and adhesion under physiological loading: although the coatings exhibit good interfacial integrity, comprehensive quantitative adhesion testing (e.g., standardized pull-off or fatigue loading tests) is required. In load-bearing dental applications, coatings must withstand cyclic mechanical stresses without delamination or degradation; (ii) long-term stability and degradation behavior: the dissolution–reprecipitation behavior of biomimetic CaP coatings is beneficial for bioactivity, but long-term studies are necessary to evaluate coating stability, ion release kinetics over extended periods, and the evolution of the coating under physiological conditions; (iii) in vivo biological performance: the current study is limited to in vitro evaluations. Animal studies are required to confirm osseointegration, bone–implant interfacial strength, inflammatory response, and antibacterial efficacy in a complex biological environment; (iv) optimization of Zn concentration: the results indicate a balance between antibacterial activity and cytocompatibility. Excessive Zn incorporation may negatively affect cell viability, highlighting the need to define an optimal therapeutic window that maximizes antimicrobial performance without compromising tissue integration; (v) surface functional uniformity and scalability: for clinical applications, the coating process must be reproducible on complex implant geometries and scalable for industrial manufacturing while maintaining uniform thickness, composition, and performance; (vi) regulatory and sterilization considerations: the effect of sterilization methods (e.g., autoclaving, gamma irradiation) on coating structure, adhesion, and ion release must be assessed. Additionally, compliance with regulatory standards for medical devices requires extensive biocompatibility and safety validation.

In summary, while the Zn–CaP coatings demonstrate strong potential as multifunctional surfaces for implant applications, further mechanical, biological, and translational studies are necessary to ensure their safety, reliability, and clinical effectiveness.

## 4. Conclusions

This study demonstrates the successful fabrication of biomimetic Zn-doped calcium phosphate (Zn–CaP) coatings on surface-activated Ti6Al4V substrates through a low-temperature route under physiologically relevant conditions. A sequential chemical–thermal activation strategy (NaOH–CaCl_2_–TT–H_2_O) effectively transformed the initially bioinert alloy into a chemically reactive, calcium-enriched titanate surface. This hydroxylated TiO_2_-based interfacial layer played a decisive role in promoting heterogeneous nucleation and strong interfacial bonding, confirming that surface chemistry (rather than roughness alone) governs the efficiency, uniformity, and adhesion of CaP coatings.

A systematic evaluation of deposition parameters identified a well-defined processing window for structurally reliable coatings. Insufficient ionic supersaturation or short immersion times resulted in discontinuous layers, whereas excessive deposition led to crack formation due to residual stresses. Among the studied conditions, the SBFX10–4 h treatment (VD3) provided the optimal balance, yielding homogeneous, crack-free coatings with high surface coverage. These findings highlight the critical role of controlling supersaturation and deposition kinetics in achieving mechanically stable and functionally reliable biomimetic coatings.

Zinc incorporation was successfully achieved and confirmed by EDS and XPS, demonstrating that Zn is present as Zn^2+^ chemically associated with the CaP phase. The results reveal that Zn incorporation occurs through a combination of structural substitution and surface-associated species, leading to a non-linear relationship between nominal Zn content and ion release behavior. This incorporation significantly modified the precipitation mechanisms and phase composition, as reflected by Ca/P ratios ranging from 1.44 to 1.80. Low and intermediate Zn levels promoted calcium-deficient apatite formation, whereas higher Zn concentrations shifted the system toward Ca-rich compositions, indicating altered crystal growth dynamics. Among the evaluated variants, VDZn2 (≈0.5–0.7 at% Zn) provided the most favorable compositional and structural balance, maintaining a Ca/P ratio closer to apatite stoichiometry while preserving coating homogeneity.

The Zn–CaP coatings exhibited a characteristic two-stage Zn^2+^ release mechanism, consisting of an initial burst release (up to 0.059 mg·L^−1^ at 24 h) followed by a sustained release reaching approximately 0.060–0.069 mg·L^−1^ after 168 h. This behavior demonstrates that biological performance is governed not only by total Zn content but also by its physicochemical state and distribution within the coating. The initial release is sufficient to induce antibacterial activity, while the subsequent controlled release supports bioactivity without exceeding cytotoxic thresholds.

From a functional standpoint, the coatings exhibited a clear, concentration-dependent antibacterial effect against *Streptococcus mutans*, with a reduction in bacterial colonies of up to ~47% compared with Zn-free CaP. At the same time, all coatings maintained cell viability above the 70% threshold defined by ISO 10993-5:2009, Annex C (*Biological Evaluation of Medical Devices—Part 5: Tests for In Vitro Cytotoxicity*; International Organization for Standardization (ISO): Geneva, Switzerland, 2009), confirming their cytocompatibility. However, increasing Zn content resulted in a moderate decrease in cell viability, highlighting the existence of a narrow therapeutic window. In this context, intermediate Zn incorporation (VDZn2) represents the most balanced condition, combining effective antibacterial activity with acceptable cytocompatibility.

Compared with conventional Zn-doped CaP or hydroxyapatite coatings produced by high-temperature or electrochemical techniques, the present biomimetic approach offers several advantages, including low processing temperature, closer structural similarity to biological apatite, and improved control over ion incorporation and release kinetics. These features are particularly relevant for the development of multifunctional implant coatings capable of simultaneously promoting osseointegration and reducing infection risk.

Despite these promising outcomes, several limitations should be acknowledged. The biological assessment was restricted to in vitro assays, and long-term stability under dynamic physiological conditions was not fully evaluated. In addition, although an optimal Zn range was identified, further refinement is required to precisely define the therapeutic window. Variability inherent to biomimetic deposition, such as local differences in thickness and microstructure, also requires further investigation.

Future work should focus on long-term in vitro and in vivo validation, quantitative mechanical adhesion and fatigue testing, and optimization of Zn distribution through controlled processing strategies. The incorporation of multiple bioactive ions, as well as the design of gradient or hierarchical coatings, represents a promising direction to further enhance multifunctional performance.

This study provides new insight into the interplay between surface activation, Zn incorporation, ion release kinetics, and biological response in biomimetic CaP coatings. The results demonstrate that controlling not only composition but also the physicochemical state of dopants is essential for tailoring coating performance, offering a rational framework for the design of next-generation multifunctional implant surfaces.

## Figures and Tables

**Table 1:** Mass of salts used to prepare 500 mL of CaP solutions and corresponding molar concentrations.

Solution	Weight of salts (g)		
	CaCl_2_	NaH_2_PO_4_	NaHCO_3_
SBFX7	0.9711	0.2625	0.1278
SBFX10	1.3873	0.3749	0.1890
	**Molar concentration of salts (mmol/L)**		
	**CaCl_2_**	**NaH_2_PO_4_**	**NaHCO_3_**
SBFX7	17.50	4.4	3.04
SBFX10	25	6.25	4.50

**Table 2:** Deposition variants evaluated in this study.

Deposition Variants	Solution	Deposition Time (h)
VD1	SBFX7	4
VD2	SBFX7	6
VD3	SBFX10	4
VD4	SBFX10	6

**Table 3:** Deposition variants with Zn^2+^ incorporation.

Deposition Variants	ZnCl_2_ (mg)	Zn^2+^ Concentration (mmol/L)
VDZn1	22	0.3
VDZn2	82	1.2
VDZn3	136	2.0

**Table 5:** Semi-quantitative point EDS analysis of Zn-containing coating variants (atomic %).

Deposition Variant	Elements (at%)							
	C	O	Al	P	Ca	Ti	Zn	Ca/P Ratio
VDZn1	21.9	55.3	0.4	4.5	6.5	3.0	0.3	1.44
VDZn2	29.5	56.4	0.4	3.9	5.9	3.4	0.5	1.51
VDZn3	26.8	58.4	0.4	3.5	6.3	4.3	0.4	1.8

**Table 7:** Semi-quantitative elemental mapping (EDS) analysis of Zn-containing coating variants (atomic %).

Deposition Variant	Elements (at%)			
	O	P	Ca	Zn
VDZn1	85.4	2.4	11.6	0.6
VDZn2	82.7	4.2	12.4	0.7
VDZn3	86.4	4.6	8.0	1.0

**Table 10:** Cumulative Zn^2+^ release (mg·L^−1^) in PBS at different incubation times.

Time (h)	VDZn1 (mg·L^−1^) ± SD	VDZn2 (mg·L^−1^) ± SD	VDZn3 (mg·L^−1^) ± SD
24	0.059 ± 0.003	0.023 ± 0.0015	0.033 ± 0.0020
72	0.081 ± 0.004	0.043 ± 0.0025	0.052 ± 0.0030
168	0.101 ± 0.006	0.060 ± 0.0035	0.069 ± 0.0040

**Table 12:** Surface roughness parameters (Ra, Rz, and Rq) of biomimetic Zn–CaP coatings deposited on Ti6Al4V substrates at different Zn concentrations. Values are expressed as means ± standard deviations (*n* = 3). Ra: arithmetic mean roughness; Rz: average maximum height of the profile; Rq: root mean square roughness.

Coating Variant	Ra (µm) ± SD	Rz (µm) ± SD	Rq (µm) ± SD
VDZn0	0.75 ± 0.07	5.39 ± 0.64	0.98 ± 0.09
VDZn1	0.50 ± 0.18	3.83 ± 0.51	0.69 ± 0.11
VDZn2	0.39 ± 0.04	2.81 ± 0.24	0.49 ± 0.04
VDZn3	0.72 ± 0.08	4.87 ± 0.30	0.95 ± 0.04

## Data Availability

The original contributions presented in the study are included in the article; further inquiries can be directed to the corresponding authors.

## Abbreviations

The following abbreviations are used in this manuscript:

SBF	Simulated body fluid
CaP	Calcium phosphate
Zn–CaP	Zn-doped calcium phosphate
HA	Ca_10_(PO_4_)_6_(OH)_2_
DMEM	Dulbecco’s Modified Eagle Medium (DMEM)
VD	Deposition Variants
BHI	Brain Heart Infusion BHI
PBS	Phosphate-buffered saline PBS
CFU	Colony-forming units
